# Novel Strategies for Assessing Associations Between Selenium Biomarkers and Cardiometabolic Risk Factors: Concentration, Visit-to-Visit Variability, or Individual Mean? Evidence From a Repeated-Measures Study of Older Adults With High Selenium

**DOI:** 10.3389/fnut.2022.838613

**Published:** 2022-05-30

**Authors:** Ang Li, Quan Zhou, Yayuan Mei, Jiaxin Zhao, Meiduo Zhao, Jing Xu, Xiaoyu Ge, Qun Xu

**Affiliations:** ^1^Department of Epidemiology and Biostatistics, Institute of Basic Medical Sciences Chinese Academy of Medical Sciences, School of Basic Medicine Peking Union Medical College, Beijing, China; ^2^Center of Environmental and Health Sciences, Chinese Academy of Medical Sciences, Peking Union Medical College, Beijing, China

**Keywords:** concentration, visit-to-visit variability (VVV), individual mean (IM), cardiometabolic risk factors, selenium

## Abstract

**Background and Aims:**

Previous studies have focused only on the cardiometabolic effects of selenium concentrations. We explored whether selenium levels and their visit-to-visit variability (VVV) and individual mean (IM) are independently associated with cardiometabolic risk factors.

**Methods:**

A three-wave repeated-measures study of older adults with high selenium (*n* = 201) was conducted in Beijing from 2016 to 2018. Whole blood selenium and urinary selenium concentrations were measured. VVV and IM were used to profile the homeostasis of the selenium biomarkers. Four indicators, namely standard deviation, coefficient of variation, average real variability, and variability independent of the mean, were employed to characterize VVV. We considered 13 cardiometabolic factors: four lipid profile indicators, three blood pressure indices, glucose, uric acid, waistline, hipline, waist-hip ratio, and sex-specific metabolic syndrome score. Linear mixed-effects regression models with random intercepts for the participants were employed to explore the associations of the selenium concentrations, VVV, and IM with the cardiometabolic factors.

**Results:**

The geometric mean whole blood and urinary selenium levels were 134.30 and 18.00 μg/L, respectively. Selenium concentrations were significantly associated with numerous cardiometabolic factors. Specifically, whole blood selenium was positively associated with total cholesterol [0.22, 95% confidence interval (CI): 0.12, 0.33], low-density lipoprotein cholesterol (LDL-C; 0.28, 95% CI: 0.13, 0.42), glucose (0.22, 95% CI: 0.10, 0.34), and uric acid (0.16, 95% CI: 0.04, 0.28). After adjustment for VVV, the IM of whole blood selenium was positively correlated with total cholesterol (0.002, 95% CI: 0.001, 0.004), triglycerides (0.007, 95% CI: 0.004, 0.011), and LDL-C (0.002, 95% CI: 0.000, 0.004). However, we did not observe any robust associations between the VVV of the selenium biomarkers and cardiometabolic risk factors after adjustment for IM.

**Conclusion:**

Our findings suggest that selenium concentrations and their IMs are significantly associated with cardiometabolic risk factors among older adults with high selenium. Longer repeated-measures studies among the general population are required to validate our findings and elucidate the relevant underlying mechanisms.

## Introduction

Cardiovascular disease (CVD) is a leading cause of death in East Asia and the world. In 2019, an estimated 18.6 million [95% confidence interval (CI): 17.1–19.7 million] CVD-related deaths occurred globally (1). Cardiometabolic risk factors, including dyslipidaemia, hypertension, diabetes, and obesity, are the primary causes of CVD (2, 3). Because of the antioxidant effects of selenium, selenium supplements and high-selenium foods are widely consumed to prevent the development of cardiometabolic risk factors (4). However, evidence on the influence of selenium on cardiometabolic risk factors remains inconclusive, and selenium may even increase cardiometabolic risk (5–7). Thus, whether selenium should be promoted for its cardiometabolic protective effects remains uncertain.

A cross-sectional study of healthy adults in two Chinese counties with different selenium intake habits indicated that serum selenium was positively correlated with serum glucose in those with selenium deficiency (median: 58 μg/L). However, no significant relationship between serum selenium and glucose was observed in those without such deficiency (median: 103 μg/L) (8). Additionally, a cross-sectional analysis using data from the National Health and Nutrition Examination Survey (NHANES) reported a U-shaped association between plasma selenium (mean: 137.1 μg/L) and the likelihood of diabetes, with the lowest risk noted for a concentration of ~122 μg/L (9). An experimental study discovered that the dose-dependent effects of selenium range from antioxidation and anti-inflammation to the promotion of oxidative stress and insulin resistance (10). Therefore, two crucial factors must be emphasized regarding the effect of selenium on cardiometabolic risk: the *U*-shaped associations between selenium levels and cardiometabolic risk factors and the broad individual variation in selenium concentrations. According to the study of Reference Man by the International Commission on Radiological Protection, a mean whole blood selenium exceeding 130 μg/L is defined as high selenium (11). However, most relevant epidemiological studies have been conducted on populations with moderate or deficient selenium levels, with means of blood selenium concentrations ranging from 47 to 127.5 μg/L (12–14). Few epidemiological studies have been conducted in populations with high selenium (>130 μg/L), limiting the understanding of the role of high selenium in the development of cardiometabolic risk factors (15, 16). Selenium-rich soil with concentrations exceeding 0.4 mg/kg has been discovered in areas across Beijing (17), providing an excellent opportunity to investigate the association between high selenium levels and cardiometabolic risk in a real-world setting. Compared with young adults, fewer older adults (aged 50 years or older) smoke or consume alcohol (18, 19). In addition, the metabolic and physiological functions, including excretion function, of older adults are gradually impaired during the aging process (20, 21). Therefore, compared with other age groups, older adults have higher selenium levels and more cases of high selenium (22–24). Moreover, aging adults experience multisystem functional impairment and increasing susceptibility to multiple chronic diseases (25). Therefore, examining older adults in a study of the cardiometabolic health effects of selenium is warranted.

Selenium assessment is mainly based on selenium concentrations in blood and urine. Increasing evidence suggests inconsistent associations among urinary selenium, circulating selenium (whole blood selenium, plasma selenium, or serum selenium), and cardiometabolic risk factors. A case–control study conducted in Wuhan, China, indicated no association between urinary selenium (mean: 20.47 μg/g) and blood pressure (BP) (26). However, another case–control study conducted in Wuhan demonstrated a U-shaped association between plasma selenium (median: 92.66 μg/L) and hypertension (12). Therefore, the associations of multiple selenium measures (e.g., whole blood and urinary selenium) with cardiometabolic risk factors should be examined simultaneously. Three cross-sectional analyses using data from the NHANES reported diverse associations of serum selenium with blood pressure and glucose and lipid profiles. *U*-shaped associations of serum selenium (mean: 137.1 μg/L) with systolic BP (SBP) and pulse pressure were observed (27). In addition, high serum selenium (mean: 137.1 μg/L) was associated with high glucose levels (9). Another study discovered that total cholesterol and triglyceride levels increased with serum selenium (median: 192.99 μg/L); furthermore, low-density lipoprotein cholesterol (LDL-C) was non-linearly associated with serum selenium, but high-density lipoprotein cholesterol (HDL-C) did not vary with serum selenium (5). This evidence suggests that multiple cardiometabolic risk factors should be measured to comprehensively characterize the associations of selenium levels with cardiometabolic health and evaluate the consistency of results to draw robust conclusions.

Changes in diet, lifestyle, or daily activities dynamically affect biomarkers and physiological parameters and these variations influence health endpoints (28). Visit-to-visit variability (VVV) in BP and glucose and lipid profiles has been associated with CVD and mortality (29–31). Selenium homeostasis is essential for a wide range of cellular functions, such as modulation of the cell cycle and apoptosis, redox balancing, and protein and DNA synthesis (32). Therefore, investigating the VVV or individual mean (IM) of selenium levels may be beneficial, while exploring the associations between selenium and cardiometabolic risk factors. Such considerations may elucidate a crucial means of reducing misclassifications in exposure assessment and further understanding the associations between selenium and cardiometabolic health. Because VVV and IM data cannot be calculated in a traditional cross-sectional study or a cohort study measuring only baseline exposure, a repeated-measures study is required.

Using a three-wave repeated-measures study of 201 older adults with high selenium residing in Beijing, China, from 2016 through 2018, we explored the associations between selenium biomarkers (i.e., whole blood and urinary selenium concentration) and cardiometabolic risk factors. On the basis of the selenium concentrations measured during three clinic visits, selenium homeostasis factors, namely VVV and IM, were considered. Furthermore, we investigated the associations of the VVV and IM of selenium levels with cardiometabolic risk factors.

## Methods

### Study Setting and Population

**Figure 1:**
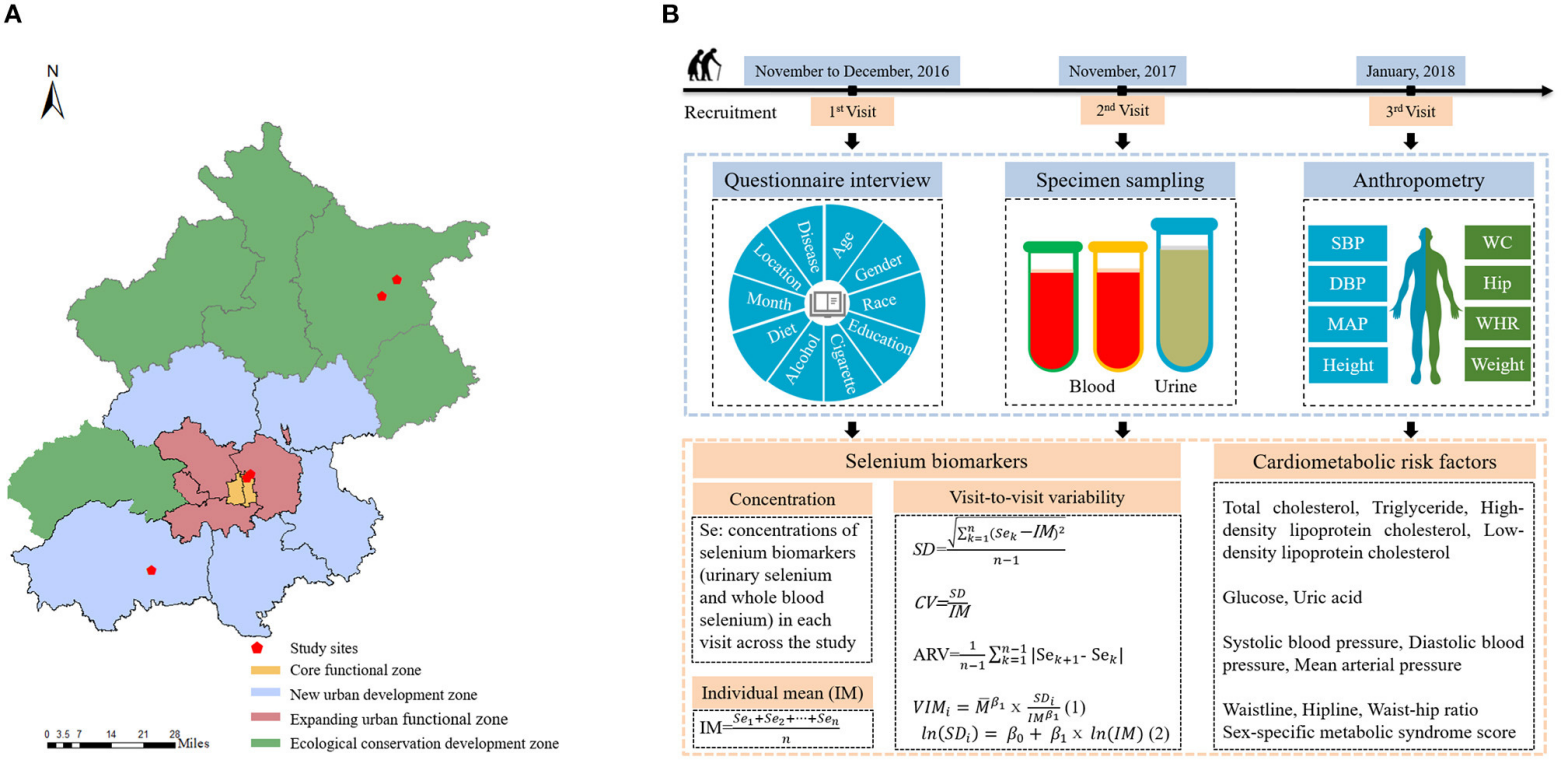
**(A)** Five study locations within Beijing. **(B)** Overview of study design and data collection.

The study was conducted in Beijing, which is in the northern North China Plain. As previously mentioned, selenium-rich soil has been discovered across the Beijing area from north to south. We selected five communities (Qian Nantai, Liu Hegou, Dongcheng, Chaoyang, and Fangshan) from four regions of Beijing from north to south to serve as the study settings. Among five communities, Qian Nantai, Liu Hegou are located in rural area. Dongcheng, Chaoyang and Fangshan are located in urban area. Further details regarding the study setting and design are presented in Figure 1.

The inclusion and exclusion criteria were the same as those described in a previous study (33). We attempted to sample a population of older adults with stable selenium statuses and the ability to complete three clinic visits by recruiting individuals aged 50 years or older who had lived in the community for more than 5 years and were unlikely to leave during the study period. Those who were unable to complete a questionnaire survey, had received a malignant tumor diagnosis, or had CVD or liver disease were excluded. In addition, a questionnaire inquired into the participants' dietary supplement intake, and the participants did not consume any dietary selenium supplements. All three measurements were conducted in the winter to control for confounding seasonal factors. After the first visit in November or December 2016, 201 participants were enrolled. Two follow-up surveys were conducted in November 2017 and January 2018. Ultimately, 83% (*n* = 167) of the study participants completed all three visits, and 17% (*n* = 34) completed only two visits. In total, 569 observations were analyzed.

The study protocol was reviewed and approved by the Institutional Ethics Committee of the Institute of Basic Medicine at the Chinese Academy of Medical Sciences. Each participant provided written consent before participating.

### Measurement of Whole Blood Selenium and Urinary Selenium

#### Species Sampling

The participants fasted overnight (≥8 h) before each examination. A qualified nurse obtained peripheral blood samples from all participants at the same time of day (8:00–9:00 am) to control for variations in circadian rhythm. Whole blood and serum were collected for whole blood selenium concentration measurements and serum biochemical analyses. First morning urine samples (10 mL) were collected from the participants in trace element—free containers. All urine and blood samples were stored at −80°C for subsequent analysis.

#### Measurements of Blood and Urine Samples

Before analysis, the whole blood samples were diluted (1:10 ratio) in a digestive solution composed of 0.5% nitric acid and 0.01% Triton X-100. The urine samples were diluted (1:10) in a digestive solution composed of 0.5% nitric acid and 0.02% Triton X-100. Both the whole blood selenium and urinary selenium concentrations were measured through inductively coupled plasma mass spectrometry (Nexion 300D, PerkinElmer, USA). In addition, we applied quality control protocols to ensure the accuracy of our analyses. First, all the samples were measured three times. Second, standard reference materials were measured every 20 samples to ensure that the whole blood and urinary selenium measurements were consistent with certified concentrations before subsequent samples were assayed. The limits of detection (LODs) for whole blood and urinary selenium were determined to be 2.15 and 0.14 μg/L, respectively. The concentrations in all samples were higher than the LODs.

#### Adjustment of Urinary Selenium Concentration

In epidemiological studies of environmental contaminants measured in urine, adjustment for creatinine to control for the effect of urine dilution is common, but the optimal approach is debated (34). Traditionally, urinary biomarker concentrations have been standardized through division by the urinary creatinine concentration, in accordance with the assumption that creatinine excretion is approximately constant across individuals and time (35, 36). However, increasing evidence suggests that creatinine levels can vary with time and individual characteristics, including sex, race, age, and body mass index (BMI); thus, conventional creatinine adjustment may yield misleading conclusions. Therefore, we employed a three-step approach, enabling systematic individual differences in long-term creatinine levels to be used to estimate covariate-adjusted, standardized urinary selenium concentrations (37). Urinary creatinine concentrations were measured using the sarcosine oxidase method. First, natural log–transformed creatinine was regressed on factors that can affect urine dilution. We included age, sex, race, weight, and height as covariates in the regression model (38). The measured creatinine concentration was divided by the fitted creatinine value obtained from the regression model to obtain a ratio representing the covariate-independent residual effect of hydration on creatinine. We finally standardized the urinary selenium concentration by dividing it by this ratio. This method specifically controls the covariate-independent, short-term multiplicative effect of hydration on urine dilution, avoids potential collider bias resulting from the use of urinary creatinine or specific gravity in adjusting for urine dilution, and increases the accuracy of exposure assessment (38, 39).

#### Calculation of VVV and IM of Selenium Biomarkers

The VVV and IM of the selenium biomarkers were calculated to estimate the fluctuation and homeostasis of the biomarkers across three visits. The IM of the selenium biomarkers for each participant was the mean selenium concentrations from the three visits. No gold standard regarding the appropriate indicators of the VVV of selenium biomarkers exists (40). According to the concentrations of the selenium biomarkers across the three visits, we calculated four indices of VVV, namely the standard deviation (SD), coefficient of variation (CV), average real variability (ARV), and variability independent of the mean (VIM), to comprehensively assess variability. SD and CV are simple and informative indices of VVV (41). SD was used to indicate the general variation between the selenium biomarker levels and the mean values of the participants. However, the order of measurement is not considered in SD calculation (42). ARV was used to overcome the limitations of SD by accounting for the order of measurements when quantifying VVV (29). However, ARV was correlated with the selenium concentrations. The CVs of the selenium biomarkers across the three visits were computed from the mean and SD. Therefore, the CVs might be correlated with the mean selenium concentrations (43). Hence, the VIM was calculated through logarithmic curve fitting to eliminate the correlation with the biomarkers, ensuring that we could evaluate the impact of the VVV separately from the effect of the selenium biomarker levels themselves (29). The formulae for the four VVV indices are as follows:


(1) $$\begin{array}{ccc} SD & = & \frac{\sqrt{\sum_{k=1}^{n}{\left(Se_{k}-IM\right)}^{2}}}{n-1} \end{array}$$

(2) $$CV\;=\;\frac{SD}{IM}$$

(3) $$ARV\;=\;\frac{1}{n-1}\;\sum_{k=1}^{n-1}\left|Se_{k+1}-Se_{k}\right|\;$$

(4-1) $$\ln(SD_{i})\;=\;\beta_{0}+\beta_{1}\ln(IM)$$

(4-2) $$VIM_{i}\;=\;{\bar{M}}^{\beta_{1}}\frac{SD_{i}}{IM^{\beta_{1}}}$$

where *Se* denotes the selenium concentration; *IM* denotes the individual mean of the concentration; *n* is the total number of measurements (*n* = 2 or 3); *k* indicates the visit number (*k* = 1, 2, or 3); *Se*_*K*_ is the selenium concentration recorded during visit *k*; $\bar{M}$ is the overall mean of the selenium concentration for the study population; *SD*_*i*_ is the SD of the selenium measurements for individual *i*; and *VIM*_*i*_ is the VIM of the selenium measurements for individual *i*. In total, we calculated eight selenium VVV indicators: the SD, CV, ARV, and VIM of whole blood and urinary selenium (denoted as SD-WBSe, CV-WBSe, ARV-WBSe, VIM-WBSe, SD-USe, CV-USe, ARV-USe, and VIM-USe, respectively).

### Outcome Assessment

#### Physical Measurement

The participants' upper arm BP was measured using calibrated mercury sphygmomanometer at least three times after the participants had rested for 15 min in a room. SBP and diastolic BP (DBP) were obtained at the appearance and disappearance of Korotkoff sounds, respectively. The interval between measurements was required to exceed 2 min. Additional measurements were taken if the differences among measurements exceeded 5 mmHg. The mean of the final two measurements was calculated for subsequent analysis. Mean arterial pressure (MAP) was calculated because MAP is associated with the risks of adverse cardiovascular events (44). The following formula was used to estimate MAP:


(5) $$\begin{array}{r} MAP=\left(2\times DBP+SBP\right)/3 \end{array}$$

Height was measured using a verified stadiometer, with each participant in a standing position with shoes removed, shoulders relaxed, head facing forwards, and back facing the wall. Weight was measured with the participants wearing little clothing, by using a certified body composition analyser. The participants' weights in kilograms were divided by their heights in meters squared to obtain their BMIs. For waistline and hipline measurements, each participant was asked to wear minimal clothing and stand with their feet close together, arms at their side, and body weight evenly distributed. Waistline was measured in centimeters at the midpoint between the lower margin of the last palpable rib and the top of the iliac crest using stretch-resistant tape. Hipline measured in centimeters around the widest portion of the buttocks, with the tape parallel to the floor (45). The waistline measurements were divided by the hipline measurements to obtain waist-hip ratios. All the measurements were performed twice and averaged for reliability and accuracy.

#### Clinical Laboratory Examination

The serum samples were sent to the Clinical Laboratory Center of Peking Union Medical College Hospital for biochemical testing. Lipid (total cholesterol, triglycerides, HDL-C, and LDL-C) and glucose, uric acid, and high-sensitivity C-reactive protein (hs-CRP) levels were measured using a Beckman Coulter analyser (AU2700, Beckman Coulter, Brea, CA, USA).

#### Metabolic Syndrome Score

The metabolic syndrome score (MSS), calculated from various factors related to metabolic syndrome, is an accurate indicator of cardiometabolic risk (46). The MSS is correlated with long-term risk for diabetes and CVD and is widely employed in clinical practice to identify patients with high such risks (47, 48). To account for potential sex-based differences in the MSSs, we computed the sex-specific MSS (MSS-sex) for each participant to comprehensively evaluate their cardiometabolic health (49). MSS-sex was calculated by summary of sex-specific standardized *Z*-scores for each of five components including MAP, HDL-C, triglyceride, waistline, and glucose, generated from the population being studied (50).

### Ascertainment of Covariates

Questionnaires were used to obtain the demographic characteristics, including age (years), sex (male or female), race (Han or others), and educational attainment (illiteracy or primary school, or junior school, or senior high school and above); lifestyle characteristics, such as cigarette smoking (current, former and never) and alcohol consumption (current, former and never) habits; and health status. The participants' communities were coded as categorical variables. Additionally, because this was a longitudinal study, controlling for temporal confounders was necessary. Therefore, sampling months were recorded and adjusted for as potential confounders.

In accordance with China's medical guidelines for the prevention and treatment of dyslipidaemia in adults (51), we considered hypercholesterolaemia, hypertriglyceridemia, hyperalphalipoproteinaemia, hyperbetalipoproteinaemia, or the use of any antidyslipidaemic agents (e.g., statins) , or both to indicate dyslipidaemia. Hypercholesterolaemia was defined as total cholesterol ≥6.2 mmol/L, hypertriglyceridemia was defined as triglycerides ≥2.3 mmol/L, hyperalphalipoproteinaemia was defined as HDL-C ≤ 1.0 mmol/L, and hyperbetalipoproteinaemia was defined as LDL-C ≥ 4.1 mmol/L. Hypertension was indicated by any antihypertensive medication prescription or BP ≥140/90 mmHg measured under standardized clinical conditions (52). Diabetes was defined as fasting glucose ≥7.0 mmol/L, 2-h glucose ≥11.1 mmol/L, or current antidiabetic medication use (both insulin or oral antidiabetic drugs) (53).

Dietary intake information was collected using a food frequency questionnaire (FFQ), which inquired into the frequency and amount of food consumed over the previous 30 days. The FFQ comprised 81 food-related items, and its reproducibility and validity were verified in a prior study (54). The daily total energy intake (kcal/day) was determined from the FFQ data and Chinese Tables of Food Composition (55).

### Statistical Analysis

#### Statistical Description

The participants' general characteristics, demographic features, health status, VVV and IM of selenium biomarkers, and cardiometabolic risk factors were documented. The categorical variables are expressed as numbers (percentages), and the continuous variables are expressed as means ± SDs. To comprehensively profile the distribution of the selenium biomarkers, geometric means and percentiles for the selenium biomarkers were calculated. In addition, distribution characteristics of selenium biomarkers by urban and rural areas were described. Unadjusted linear mixed-effects regression models of the selenium biomarkers by area (urban area, rural area) were developed to assess difference of selenium biomarkers concentrations between two areas.

The variability in the selenium biomarkers and cardiometabolic risk factors during the study period was also calculated. We calculated intraclass correlation coefficients (ICCs) of the selenium biomarkers, defined as the ratio of the interindividual variance to the total variance, and corresponding 95% CIs. The ICCs were used to evaluate the reproducibility of the selenium biomarkers across the three visits as follows: 0.75 ≤ ICC ≤ 1.00 suggested excellent reproducibility, 0.40 ≤ ICC <0.75 suggested fair to good reproducibility, and 0.00 ≤ ICC <0.40 suggested poor reproducibility (56, 57). The distributions of the selenium biomarker values across the three visits were recorded and represented using violin plots. Spearman correlation coefficients between each pair of biomarkers measured at each visit were calculated. Unadjusted linear mixed-effects regression models of the selenium biomarkers and cardiometabolic risk factors by visit (modeled as a nominal categorical variable) were developed to assess global significance for each visit. The variability of the selenium biomarkers and cardiometabolic risk factors was helpful for both constructing models and explaining results in the statistical analyses.

#### Associations of Selenium Biomarkers With Cardiometabolic Risk Factors

##### Covariate Selection

We applied a directed acyclic graph to determine covariates adjusted in the models a prior, which was the minimum adjustment sets selected by graphical criteria such as the so-called “back-door” (58, 59). The covariates included were age, sex, race, educational attainment, cigarette smoking habits, alcohol consumption habits, BMI, sampling month, community, and daily total energy intake.

##### Exposure–Response Curves

The generalized additive mixed models (GAMMs) with embedded restricted cubic spline smoothers with three degrees of freedom were conducted to plotted exposure–response curves for the selenium biomarkers and cardiometabolic risk factors. The results of GAMMs were summarized to provide more numeric information about associations between selenium biomarkers and cardiometabolic risk factors. The linear and non-linear trends illustrated by the curves also helped guide the development and interpretation of the main analysis.

##### Main Analysis

To account for potentially skewed distributions, the concentrations of whole blood and urinary selenium were natural log–transformed before statistical analysis. Regarding outcomes, the levels of the four lipid indicators and of glucose and uric acid were natural log–transformed to reduce skewness in further analysis. Associations between the selenium biomarkers and cardiometabolic risk factors were assessed using linear mixed-effects regression models with participant-specific random intercepts to account for within-participant correlation in this repeated-measures study. For each model, one selenium biomarker was regressed on one cardiometabolic risk factor at the same study visit. According to the distributions of the selenium biomarkers and the non-linear and linear associations between them and the cardiometabolic risk factors, the selenium concentrations were included in the models as natural log–transformed continuous variables or categorical variables indicating tertiles, respectively. Linear trend *p*-values were derived by modeling the median of each selenium biomarker tertile as a continuous variable in the adjusted models. To address the multiple testing problem, in which the more inferences are made, the more likely erroneous inferences become, and reduce the probability of type I error, we adjusted the raw *p*-values on the basis of the false discovery rate (60).

##### Stratified Analysis

To account for the residual confounding and effect modification of the main analysis, we conducted analyses stratified by area (urban area or rural area), comorbidities (dyslipidaemia, diabetes, and hypertension), demographic characteristics [age (<65 or ≥65 years) and sex (male or female)], and BMI (<24 or ≥24 kg/m^2^), while simultaneously controlling for the same covariates as in the main analysis. The differences between strata were examined by estimating values and 95% CIs as follows (61).


(6) $$(\beta 1-\beta_{2})\pm 1.96\;\times \;\sqrt{{\left(SE_{1}\right)}^{2}+{\left(SE_{2}\right)}^{2}}$$

where β_1_ and β_2_ are the effect estimates attributed to each subgroup or stratum (e.g., the effects for participants with and without dyslipidaemia, respectively) and *SE*_1_ and *SE*_2_ are the corresponding standard errors. In the stratified analysis, the selenium concentrations were included in the models as natural log–transformed continuous variables.

##### Sensitivity Analysis

We conducted several sensitivity analyses to assess the robustness of the findings in the main analysis. First, to address any residual confounding attributed to chronic disease status, we additionally adjusted for history of hypertension, diabetes, or dyslipidaemia in the same model of main analysis. Second, blood selenium concentrations reportedly differ significantly with BMI (62), and a higher BMI increases the risk of cardiometabolic disease (63). Therefore, we repeated our analysis while excluding BMI from the covariates to elucidate the impact of BMI on the associations. Third, hs-CRP, an indicator of systemic inflammation, may also influence both selenium levels and cardiometabolic risk (64, 65). Therefore, we additionally adjusted for hs-CRP in the sensitivity analysis to explore its influence on the associations. In the sensitivity analysis, the selenium concentrations were incorporated into the models as natural log–transformed continuous variables.

#### Associations of VVV and IM of Selenium Biomarkers With Cardiometabolic Risk Factors

The linear mixed-effects regression models employed in the main analysis were fitted to assess the associations of the VVV and IM of the selenium biomarkers with the cardiometabolic risk factors. A single-factor models (Model 1) respectively including individual indicator of VVV or IM in selenium biomarkers were used to assess the associations of the VVV or IM of the selenium biomarkers with the cardiovascular risk factors. Furthermore, mutually adjusted models (Model 2) considering both the VVV and IM of the selenium biomarkers were used to evaluate whether the effects of VVV in selenium biomarkers on cardiovascular risk factors are independent of IM. Both models were adjusted for the same covariates as described for the main analysis.

All statistical tests were two sided, and significance was set at *p* < 0.05. All analyses were conducted using the lmerTest, gamm4, splines, corrplot, dagitty, and ggplot2 packages in R (version 4.1.0).

### Quality Assurance and Control

Standard operating procedures were followed throughout the research project. The interviewers, nurses, and physicians were all trained prior to the fieldwork. To ensure that the questionnaires collected personal information accurately, the principal investigators surveyed the interviewers with the same questionnaires to measure consistency. When each visit commenced, all the related data were recorded by qualified and trained staff (i.e., physicians, registered nurses, or trained interviewers). Data cleaning, quality assessment, and processing were performed independently by two qualified statistical analysts after completion of the surveys.

## Results

### Participant Characteristics

A summary of the participants' demographic data is presented in Table 1. Among the 201 participants included in the study, 125 (62.2 %) were female. The mean (SD) age and BMI of the participants were 64.60 (9.10) years and 25.10 (3.50) kg/m^2^, respectively. The distributions of whole blood and urinary selenium concentration across the study period are detailed in Table 2. The geometric means (geometric SDs) of urinary and whole blood selenium were 18.00 (1.87) and 134.30 (1.19) μg/L, respectively. The ICCs (95% CIs) for whole blood and urinary selenium were 0.54 (0.44, 0.61) and 0.12 (0.02, 0.22), respectively, indicating good reproducibility of the whole blood measurements and poor reproducibility of the urinary measurements (Table 2). The characteristics of the cardiometabolic risk factors and the VVV and IM of the selenium biomarkers of the participants are presented in Table 3. The means (SDs) of the SD-WBSe, ARV-WBSe, and VIM-WBSe in population were 14.00 (8.70), 17.10 (12.90), 14.00 (8.10) μg/L, respectively. By comparison, the means (SD) of the SD-USe, ARV-USe, and VIM-USe in population were lower at 11.90 (12.90), 14.30 (15.80), and 10.80 (5.00) μg/L, respectively. By contrast, the mean (SD) of the CV-USe was higher than CV-WBSe in population [0.48 (0.26) vs. 0.10 (0.06)]. Furthermore, the mean (SD) of IM-WBSe and IM-USe in population were 136.00 (20.20) and 22.30 (11.60) μg/L, respectively.

**Table 1:** Demographic characteristics and health status of participants throughout the study period.

**Variables**	**Mean ±SD or *n* (%)**
Participants	201
Age, years	64.60 ± 9.10
BMI, kg/m^2^	25.10 ± 3.50
**Gender**	
Male	76 (37.8)
Female	125 (62.2)
**Race**	
Han	195 (97.0)
Others	6 (3.0)
**Educational attainment**	
Illiteracy or primary school	67 (33.4)
Junior school	63 (31.3)
Senior high school and above	71 (35.3)
**Cigarette smoking**	
Current	25 (12.4)
Former	22 (10.9)
Never	154 (76.6)
**Alcohol consumption**	
Current	53 (26.4)
Former	13 (6.5)
Never	135 (67.2)
Total energy intake, kcal/day	1,810 ± 529
hs-CRP, mg/L	1.95 ± 2.81
**Dyslipidemia**	
Yes	101 (50.2)
No	100 (49.8)
**Hypertension**	
Yes	114 (56.7)
No	87 (43.3)
**Diabetes**	
Yes	35 (17.4)
No	166 (82.6)

*BMI, body mass index; hs-CRP, high-sensitivity C-reactive protein*.

**Table 2:** Limits of detection, distribution characteristics, and reproducibility of the selenium biomarkers.

**Analyte (μg/L)**				**Percentiles of seleniu**					**ICCs (95% CI)**
	**LOD**	**GM**	**GSD**	**5th**	**25th**	**50th**	**75th**	**95th**	
Whole blood selenium	2.15	134.30	1.19	103.90	120.40	132.10	150.40	180.00	0.54 (0.44, 0.61)
Urinary selenium	0.14	18.00	1.87	7.06	11.49	16.96	27.92	52.33	0.12 (0.02, 0.22)

*LOD, Limit of detection; GM, geometric mean; GSD, geometric standard deviation; ICCs, intraclass correlation coefficients; 95% CI, 95% confidence interval*.

**Table 3:** Summary of cardiometabolic risk factors and selenium homeostasis of participants.

**Variables**	**Mean ±SD**
**Cardiometabolic risk factors**
Total cholesterol (mmol/L)	4.96 ± 1.04
Triglyceride (mmol/L)	1.54 ± 1.08
HDL-C (mmol/L)	1.32 ± 0.33
LDL-C (mmol/L)	3.03 ± 0.86
Glucose (mmol/L)	6.07 ± 1.84
Uric acid (μmol/L)	299.00 ± 77.30
SBP (mmHg)	138.00 ± 17.70
DBP (mmHg)	83.90 ± 10.30
MAP (mmHg)	102.00 ± 11.40
Waistline (cm)	90.50 ± 9.60
Hipline (cm)	103.00 ± 7.00
Waist-hip ratio	0.88 ± 0.06
MSS-sex	0.01 ± 2.90
**Visit to visit variability indicator of selenium biomarkers**
SD-WBSe (μg/L)	14.00 ± 8.70
CV-WBSe	0.10 ± 0.06
ARV-WBSe (μg/L)	17.10 ± 12.90
VIM-WBSe (μg/L)	14.00 ± 8.10
SD-USe (μg/L)	11.90 ± 12.90
CV-Use	0.48 ± 0.26
ARV-USe (μg/L)	14.30 ± 15.80
VIM-USe (μg/L)	10.80 ± 5.00
**Individual mean indicator of selenium biomarkers**
IM-WBSe (μg/L)	136.00 ± 20.20
IM -USe (μg/L)	22.30 ± 11.60

*HDL-C, high-density lipoprotein cholesterol; LDL-C, low-density lipoprotein cholesterol; SBP, systolic blood pressure; DBP, diastolic blood pressure; MAP, mean arterial pressure; MSS-sex, metabolic syndrome score (sex specific); IM -WBSe, individual mean of whole blood selenium; SD-WBSe, standard deviation of whole blood selenium; CV-WBSe, the coefficient of variation of whole blood selenium; ARV-WBSe, average real variability of whole blood selenium; VIM-WBSe, variability independent of the mean of whole blood selenium; IM -USe, individual mean of urinary selenium; SD-USe, standard deviation of urinary selenium; CV-USe, the coefficient of variation of urinary selenium; ARV-USe, average real variability of urinary selenium; VIM-USe, variability independent of the mean of urinary selenium*.

The variations in the selenium biomarkers and cardiometabolic risk factors across the study period were also assessed, revealing three key findings: First, the global significance test revealed that whole blood selenium and most cardiometabolic risk factors (except SBP) significantly differed across the three visits (Supplementary Tables 1, 2). The difference of selenium biomarkers concentrations between urban and rural area was significant (Supplementary Table 3). Second, the overall correlation between the urinary selenium concentrations across three visits was negligible to moderate (*r*_*s*_: −0.09 to 0.56). With respect to whole blood selenium, the moderate positive correlations were found between whole blood selenium levels across three visits (*r*_*s*_: 0.61 to 0.65) which was consistent with better reproducibility of whole blood selenium compared to urinary selenium. Furthermore, there were negligible to low positive correlations between whole blood selenium concentrations and urinary selenium concentrations across three visits (*r*_*s*_: 0.07 to 0.43; Supplementary Figure 2). Final, greater variance in the distribution of whole blood selenium than that of urinary selenium was also observed, as indicated in Supplementary Figure 3.

### Associations of Selenium Biomarker Concentrations With Cardiometabolic Risk Factors

#### Exposure–Response Curves

The exposure–response curves of the selenium biomarkers and cardiometabolic risk factors obtained from the GAMMs are presented in Supplementary Figures 4, 5. Whole blood selenium was linearly associated with total cholesterol, triglycerides, HDL-C, uric acid, SBP, waistline, hipline and MSS-sex but non-linearly associated with LDL-C, glucose, DBP, MAP, and waist-hip ratio (Supplementary Figure 4). Consist with the exposure–response curves, estimated degrees of freedom (EDF) of LDL-C, glucose, DBP, MAP, and waist-hip ratio were 2.16, 2.14, 1.55, 1.46, and 2.22 which were significantly higher than 1 (Supplementary Table 4). Urinary selenium was linearly associated with cardiometabolic risk factors, except for waistline (Supplementary Figure 5). Similarly, EDF of waistline were 1.62 (Supplementary Table 4). Because of the diverse linear and non-linear relations of the selenium biomarkers with the various cardiometabolic risk factors, the selenium biomarkers were respectively considered continuous variables and categorized into tertiles in the linear mixed-effects regression models.

#### Main Analysis

In this three-wave repeated-measures study of 201 older adults with high selenium levels, we discovered that selenium levels were significantly associated with cardiometabolic risk factors after correction for multiple comparisons and adjustment for age, sex, race, educational attainment, cigarette smoking habits, alcohol consumption habits, BMI, sampling month, community, and total daily energy intake (Table 4). Primarily positive associations were observed with whole blood selenium: whole blood selenium was positively associated with total cholesterol (0.22, 95% CI: 0.12, 0.33), LDL-C (0.28, 95% CI: 0.13, 0.42), glucose (0.22, 95% CI: 0.10, 0.34), and uric acid (0.16, 95% CI: 0.04, 0.28) levels. These positive associations remained significant even with whole blood selenium concentration treated as a categorical variable (*p* trend < 0.01 for total cholesterol, LDL-C, and glucose; *p* trend = 0.02 for uric acid). Similarly, the results of GAMMs showed that associations between whole blood selenium and total cholesterol, LDL-C, glucose and uric acid were significant.

**Table 4:** Associations of selenium biomarkers with cardiometabolic risk factors, analyzed using linear mixed-effects regression models.

**Cardiometabolic risk factors**	**Regression coefficients (95% CI) by tertiles of** **selenium biomarkers concentrations**			**Per one-unit increase of selenium biomarkers^a^**	***p*** **-trend**	***p*** **-value^b^**
	**T1**	**T2**	**T3**			
**Total cholesterol**						
WBSe	Ref	**0.04 (0.01, 0.07)**	**0.08 (0.04, 0.12)**	**0.22 (0.12, 0.33)**	**<0.01**	**<0.01**
USe	Ref	0.02 (−0.01, 0.05)	0.02 (−0.01, 0.05)	0.01 (−0.02, 0.03)	0.25	0.64
**Triglyceride**						
WBSe	Ref	0.04 (−0.05, 0.14)	0.05 (−0.06, 0.18)	0.29 (0.00, 0.63)	0.39	0.08
USe	Ref	0.05 (−0.04, 0.13)	−0.01 (−0.1, 0.09)	0.01 (−0.05, 0.08)	0.96	0.76
**HDL-C**						
WBSe	Ref	0.02 (−0.01, 0.05)	0.04 (0.00, 0.07)	0.08 (−0.02, 0.19)	0.07	0.12
USe	Ref	0.01 (−0.01, 0.04)	0.03 (0.00, 0.06)	0.01 (−0.01, 0.03)	0.06	0.18
**LDL-C**						
WBSe	Ref	**0.05 (0.01, 0.09)**	**0.10 (0.05, 0.15)**	**0.28 (0.13, 0.42)**	**<0.01**	**<0.01**
USe	Ref	0.00 (−0.04, 0.04)	0.01 (−0.04, 0.05)	0.00 (−0.03, 0.03)	0.80	0.84
**Glucose**						
WBSe	Ref	**0.03 (0.00, 0.06)**	**0.07 (0.02, 0.11)**	**0.22 (0.10, 0.34)**	**<0.01**	**<0.01**
USe	Ref	−0.01 (−0.04, 0.02)	−0.03 (−0.06, 0.01)	−0.01 (−0.04, 0.01)	0.17	0.28
**Uric acid**						
WBSe	Ref	0.02 (−0.02, 0.05)	**0.05 (0.01, 0.10)**	**0.16 (0.04, 0.28)**	**0.02**	**<0.01**
USe	Ref	0.01 (−0.02, 0.05)	−0.02 (−0.05, 0.02)	−0.01 (−0.04, 0.01)	0.48	0.37
**SBP**						
WBSe	Ref	1.51 (−1.84, 4.73)	−1.25 (−5.36, 2.92)	−7.40 (−17.93, 3.40)	0.63	0.19
USe	Ref	−1.94 (−5.24, 1.26)	−3.22 (−6.82, 0.36)	**−3.37 (−5.68, −1.01)**	0.08	**<0.01**
**DBP**						
WBSe	Ref	0.47 (−1.45, 2.25)	2.06 (−0.31, 4.28)	2.64 (−3.33, 8.30)	0.09	0.39
USe	Ref	−0.51 (−2.32, 1.30)	−1.56 (−3.59, 0.42)	**−1.69 (−3.00, −0.39)**	0.14	**0.01**
**MAP**						
WBSe	Ref	1.03 (−1.08, 3.00)	0.92 (−1.68, 3.45)	−1.35 (−7.97, 5.21)	0.47	0.70
USe	Ref	−0.80 (−2.83, 1.17)	−2.17 (−4.40, 0.03)	**−2.32 (−3.76, −0.88)**	0.06	**<0.01**
**Waistline**						
WBSe	Ref	0.32 (−1.19, 1.81)	0.33 (−1.50, 2.23)	−1.40 (−5.98, 3.66)	0.73	0.58
USe	Ref	0.20 (−1.32, 1.58)	0.34 (−1.39, 1.83)	0.30 (−0.81, 1.28)	0.67	0.58
**Hipline**						
WBSe	Ref	−1.02 (−2.12, 0.07)	−0.57 (−1.98, 0.79)	−0.68 (−4.33, 2.93)	0.38	0.72
USe	Ref	0.28 (−0.79, 1.31)	0.26 (−0.95, 1.37)	0.00 (−0.77, 0.74)	0.65	0.99
**Waist-hip ratio**						
WBSe	Ref	0.01 (0.00, 0.02)	0.01 (0.00, 0.03)	0.00 (−0.04, 0.04)	0.18	0.97
USe	Ref	0.00 (−0.02, 0.01)	0.00 (−0.02, 0.01)	0.00 (−0.01, 0.01)	0.87	0.69
**MSS-sex**						
WBSe	Ref	0.30 (−0.12, 0.74)	0.28 (−0.26, 0.87)	0.44 (−1.01, 2.05)	0.30	0.58
USe	Ref	0.00 (−0.42, 0.41)	−0.29 (−0.74, 0.16)	−0.28 (−0.57, 0.02)	0.23	0.06

*95% CI, 95% confidence interval; T1, first tertile; T2, second tertile; T3, third tertile; HDL-C, high-density lipoprotein cholesterol; LDL-C, low-density lipoprotein cholesterol; SBP, systolic blood pressure; DBP, diastolic blood pressure; MAP, mean arterial pressure; MSS, metabolic syndrome score; MSS -sex, metabolic syndrome score (sex specific); WBSe, whole blood selenium; USe, urinary selenium. Boldface type indicates effect estimates were statistically significant, p-values < 0.05*.

a *Per one-unit increase of natural log transformed urinary selenium concentration or per one-unit increase of natural log transformed whole blood selenium concentration*.

b *p-values were FDR corrected*.

For urinary selenium, only negative associations were observed and only with BP-related factors. Urinary selenium was negatively associated with SBP (−3.37, 95% CI: −5.68, −1.01), DBP (−1.69, 95% CI: −3.00, −0.39), and MAP (−2.32, 95% CI: −3.76, −0.88). However, these negative associations were non-significant when urinary selenium concentration was treated as a categorical variable (*p* trend = 0.08, 0.14, and 0.06 for SBP, DBP, and MAP, respectively). The results of GAMMs showed that associations between urinary selenium and BP indicators were significant.

#### Stratified Analyses

We conducted analyses stratified by area, comorbidities (dyslipidaemia, diabetes, and hypertension status) as well as by age, sex, and BMI (Supplementary Figures 6, 7). The associations of whole blood selenium with cardiometabolic risk factors did not differ significantly with area, BMI or dyslipidaemia status but differed significantly with sex, age, diabetes status, and hypertension status (Supplementary Figure 6). The associations of urinary selenium with cardiometabolic risk factors did not differ significantly with area, age, sex, or BMI but differed significantly with dyslipidaemia, diabetes, and hypertension status (Supplementary Figure 7).

#### Sensitivity Analyses

The sensitivity analyses controlling for a history of hypertension, diabetes, or dyslipidaemia (Supplementary Figure 8) and for hs-CRP yielded results similar to those of our main analysis (Supplementary Figure 10). A significant positive association between whole blood selenium and triglyceride levels was also discovered after BMI was excluded from the covariates (Supplementary Figure 9).

### Associations Between VVV and IM of Selenium Biomarkers and Cardiometabolic Risk Factors

We observed that SD-WBSe was positively correlated with triglycerides (0.009, 95% CI: 0.000, 0.017) and MSS-sex (0.047, 95% CI: 0.005, 0.088). In addition, ARV-WBSe was positively associated with glucose (0.003, 95% CI: 0.000, 0.005) and MSS-sex (0.034, 95% CI: 0.006, 0.061) in Model 1. However, these associations were non-significant after additional adjustments for the impact of IM in Model 2. Only a marginal association of ARV-WBSe with total cholesterol (−0.003, 95% CI: −0.005, −0.000) was observed in Model 2 (Table 5).

**Table 5:** Changes and 95% confidence intervals of cardiometabolic risk factors associated with one-unit increments in VVV and IM of whole blood selenium.

	**Total cholesterol**	**Triglyceride**	**HDL-C**	**LDL-C**	**Glucose**
**Mean-WBSe**	
Model 1	**0.002 (0.001, 0.004)**	**0.007 (0.004, 0.011)**	0.001 (−0.001, 0.003)	**0.002 (0.000, 0.004)**	**0.002 (0.000, 0.004)**
Model 2	
+SD-USe	**0.003 (0.001, 0.004)**	**0.007 (0.003, 0.011)**	0.001 (−0.000, 0.003)	**0.003 (0.000, 0.005)**	**0.002 (0.000, 0.004)**
+CV-USe	**0.002 (0.001, 0.004)**	**0.007 (0.004, 0.011)**	0.001 (−0.001, 0.003)	**0.002 (0.000, 0.004)**	**0.002 (0.000, 0.004)**
+ARV-USe	**0.003 (0.001, 0.004)**	**0.007 (0.003, 0.011)**	0.001 (−0.000, 0.003)	**0.003 (0.001, 0.005)**	0.002 (−0.000, 0.004)
+VIM-USe	**0.002 (0.001, 0.004)**	**0.007 (0.004, 0.011)**	0.001 (−0.001, 0.003)	**0.002 (0.000, 0.004)**	**0.002 (0.000, 0.004)**
**SD-WBSe**	
Model 1	−0.001 (−0.004, 0.002)	**0.009 (0.000, 0.017)**	−0.002 (−0.006, 0.001)	−0.001 (−0.006, 0.003)	0.003 (−0.001, 0.007)
Model 2	−0.003 (−0.006, 0.001)	0.004 (−0.004, 0.012)	−0.003 (−0.007, 0.001)	−0.003 (−0.008, 0.002)	0.002 (−0.002, 0.006)
**CV-WBSe**	
Model 1	−0.264 (−0.744, 0.216)	0.726 (−0.484, 1.935)	−0.414 (−0.971, 0.143)	−0.306 (−0.984, 0.372)	0.218 (−0.355, 0.791)
Model 2	−0.328 (−0.798, 0.143)	0.518 (−0.649, 1.686)	−0.444 (−1.000, 0.112)	−0.370 (−1.044, 0.303)	0.157 (−0.410, 0.725)
**ARV-WBSe**	
Model 1	−0.002 (−0.004, 0.001)	0.006 (0.000, 0.011)	−0.002 (−0.004, 0.001)	−0.002 (−0.005, 0.001)	**0.003 (0.000, 0.005)**
Model 2	**−0.003 (−0.005, −0.000)**	0.003 (−0.002, 0.008)	−0.002 (−0.005, 0.000)	−0.003 (−0.006, −0.000)	0.002 (−0.001, 0.005)
**VIM-WBSe**	
Model 1	−0.002 (−0.005, 0.002)	0.005 (−0.003, 0.014)	−0.003 (−0.007, 0.001)	−0.002 (−0.007, 0.003)	0.002 (−0.003, 0.006)
Model 2	−0.002 (−0.006, 0.001)	0.004 (−0.005, 0.012)	−0.003 (−0.007, 0.001)	−0.003 (−0.008, 0.002)	0.001 (−0.003, 0.005)
	**Uric acid**	**SBP**	**DBP**	**MAP**	**Waistline**
**Mean-WBSe**					
Model 1	0.001 (−0.000, 0.003)	0.003 (−0.110, 0.114)	0.016 (−0.042, 0.072)	0.010 (−0.060, 0.078)	0.032 (−0.019, 0.079)
Model 2					
+SD-USe	0.001 (−0.001, 0.003)	−0.016 (−0.136, 0.099)	0.003 (−0.058, 0.063)	−0.003 (−0.077, 0.068)	0.032 (−0.022, 0.080)
+CV-USe	0.001 (−0.000, 0.003)	−0.002 (−0.116, 0.108)	0.013 (−0.046, 0.069)	0.007 (−0.063, 0.074)	0.032 (−0.020, 0.078)
+ARV-USe	0.001 (−0.001, 0.003)	−0.015 (−0.132, 0.100)	0.010 (−0.051, 0.068)	0.001 (−0.071, 0.071)	0.034 (−0.019, 0.082)
+VIM-USe	0.001 (−0.000, 0.003)	−0.003 (−0.117, 0.108)	0.012 (−0.046, 0.069)	0.006 (−0.064, 0.074)	0.032 (−0.020, 0.078)
**SD-WBSe**					
Model 1	0.003 (−0.000, 0.007)	0.125 (−0.121, 0.370)	0.090 (−0.037, 0.215)	0.093 (−0.058, 0.243)	0.023 (−0.085, 0.127)
Model 2	0.003 (−0.001, 0.006)	0.136 (−0.121, 0.395)	0.088 (−0.045, 0.220)	0.095 (−0.063, 0.254)	0.002 (−0.110, 0.113)
**CV-WBSe**					
Model 1	0.431 (−0.085, 0.945)	19.998 (−16.149, 56.358)	12.575 (−6.142, 31.069)	14.066 (−8.242, 36.300)	1.079 (−14.713, 16.644)
Model 2	0.395 (−0.120, 0.911)	20.056 (−16.163, 56.583)	12.248 (−6.513, 30.824)	13.890 (−8.468, 36.234)	0.201 (−15.602, 15.928)
**ARV-WBSe**					
Model 1	0.002 (−0.001, 0.004)	0.090 (−0.072, 0.252)	0.038 (−0.046, 0.121)	0.049 (−0.051, 0.149)	0.002 (−0.069, 0.070)
Model 2	0.001 (−0.001, 0.004)	0.096 (−0.071, 0.264)	0.035 (−0.052, 0.121)	0.049 (−0.054, 0.152)	−0.011 (−0.083, 0.061)
**VIM-WBSe**					
Model 1	0.003 (−0.001, 0.007)	0.146 (−0.118, 0.413)	0.093 (−0.044, 0.228)	0.103 (−0.060, 0.266)	0.009 (−0.107, 0.123)
Model 2	0.003 (−0.001, 0.007)	0.147 (−0.119, 0.415)	0.090 (−0.048, 0.226)	0.102 (−0.062, 0.266)	−0.000 (−0.001, 0.000)
	**Hipline**	**Waist-hip ratio**	**MSS_sex**		
**Mean-WBSe**					
Model 1	0.013 (−0.029, 0.050)	0.000 (−0.000, 0.001)	**0.022 (0.002, 0.041)**		
Model 2					
+SD-USe	0.007 (−0.037, 0.046)	0.000 (−0.000, 0.001)	0.017 (−0.004, 0.036)		
+CV-USe	0.011 (−0.030, 0.048)	0.000 (−0.000, 0.001)	**0.021 (0.001, 0.039)**		
+ARV-USe	0.008 (−0.035, 0.047)	0.000 (−0.000, 0.001)	0.017 (−0.003, 0.036)		
+VIM-USe	0.011 (−0.031, 0.048)	0.000 (−0.000, 0.001)	**0.021 (0.001, 0.039)**		
**SD-WBSe**					
Model 1	0.046 (−0.040, 0.131)	−0.000 (−0.001, 0.001)	**0.047 (0.005, 0.088)**		
Model 2	0.042 (−0.048, 0.132)	−0.000 (−0.001, 0.000)	0.035 (−0.008, 0.079)		
**CV-WBSe**					
Model 1	6.414 (−6.285, 18.984)	−0.043 (−0.158, 0.071)	5.099 (−1.121, 11.285)		
Model 2	6.115 (−6.600, 18.806)	−0.049 (−0.164, 0.065)	4.511 (−1.674, 10.695)		
**ARV-WBSe**					
Model 1	0.028 (−0.029, 0.084)	−0.000 (−0.001, 0.000)	**0.034 (0.006, 0.061)**		
Model 2	0.025 (−0.033, 0.084)	−0.000 (−0.001, 0.000)	0.028 (−0.001, 0.056)		
**VIM-WBSe**					
Model 1	0.047 (−0.046, 0.139)	−0.000 (−0.001, 0.001)	0.038 (−0.008, 0.083)		
Model 2	0.045 (−0.048, 0.138)	0.002 (−0.114, 0.117)	0.033 (−0.012, 0.079)		

*Boldface type indicates effect estimates were statistically significant, p-values < 0.05*.

Consistent with results of the main analysis, the IM of whole blood selenium was positively correlated with total cholesterol (0.002, 95% CI: 0.001, 0.004), triglycerides (0.007, 95% CI: 0.004, 0.011), and LDL-C (0.002, 95% CI: 0.000, 0.004) in Model 1, and these associations remained significant after adjustment for the impact of VVV in Model 2. The positive association between the IM of whole blood selenium and glucose (0.002, 95% CI: 0.000, 0.004) was also observed in Model 1, and this association remained significant after adjustment for SD-WBSe, CV-WBSe, and VIM-WBSe in Model 2. Furthermore, we discovered a positive association between the IM of whole blood selenium and MSS-sex (0.022, 95% CI: 0.002, 0.041) in Model 1, and this association was significant after adjustment for CV-WBSe and VIM-WBSe in Model 2 (Table 5).

We did not observe any robust associations of the VVV of urinary selenium with cardiometabolic risk factors, discovering only a marginal association of SD-USe with total cholesterol (−0.004, 95% CI: −0.007, −0.000) after adjusting for the IM of urinary selenium in Model 2. The IM of urinary selenium was positively related to triglycerides (0.014, 95% CI: 0.002, 0.025) and glucose (0.006, 95% CI: 0.000, 0.011) levels after adjustment for SD-USe in Model 2. Although urinary selenium concentration was negatively associated with SBP, DBP, and MAP in the main analysis, the IM of urinary selenium was not associated with SBP, DBP, or MAP. After additional adjustment for VIM-USe in Model 2, the estimated strengths of the associations of the IM of urinary selenium with SBP, DBP, and MAP were −0.029 (95% CI: −0.267, 0.208), −0.050 (95% CI: −0.175, 0.076), and −0.040 (95% CI: −0.188, 0.109), respectively. A negative association between the IM of urinary selenium and waistline (−0.105, 95% CI: −0.195, −0.010) was also discovered, and the association remained significant after adjustment for CV-USe, ARV-Use, and VIM-USe in Model 2. Moreover, a higher IM of urinary selenium was associated with a smaller hipline (−0.085, 95% CI: −0.161, −0.005) after additional adjustment for VIM-USe in Model 2 (Table 6).

**Table 6:** Changes and 95% confidence intervals of cardiometabolic risk factors associated with one-unit increments in VVV and IM of urinary selenium.

	**Total cholesterol**	**Triglyceride**	**HDL-C**	**LDL-C**	**Glucose**
**Mean-USe**					
Model 1	0.001 (−0.002, 0.004)	0.006 (−0.001, 0.014)	0.000 (−0.003, 0.004)	0.000 (−0.004, 0.004)	0.003 (−0.000, 0.007)
Model 2					
+SD-USe	0.005 (−0.000, 0.009)	**0.014 (0.002, 0.025)**	0.002 (−0.003, 0.007)	0.003 (−0.003, 0.010)	**0.006 (0.000, 0.011)**
+CV-USe	0.002 (−0.001, 0.005)	0.008 (−0.000, 0.016)	0.001 (−0.002, 0.005)	0.000 (−0.004, 0.005)	0.004 (−0.000, 0.008)
+ARV-USe	0.004 (−0.001, 0.008)	0.010 (−0.000, 0.021)	0.002 (−0.003, 0.007)	0.002 (−0.004, 0.008)	0.005 (−0.000, 0.010)
+VIM-Use	0.001 (−0.002, 0.004)	0.006 (−0.002, 0.014)	0.001 (−0.003, 0.005)	−0.000 (−0.005, 0.004)	0.003 (−0.001, 0.007)
**SD-USe**					
Model 1	−0.001 (−0.004, 0.001)	−0.001 (−0.006, 0.005)	0.000 (−0.003, 0.003)	−0.002 (−0.005, 0.001)	0.000 (−0.003, 0.003)
Model 2	**−0.004 (−0.007, −0.000)**	−0.008 (−0.016, 0.000)	−0.001 (−0.005, 0.003)	−0.004 (−0.009, 0.001)	−0.003 (−0.007, 0.001)
**CV-USe**					
Model 1	−0.091 (−0.204, 0.021)	−0.180 (−0.459, 0.101)	−0.044 (−0.173, 0.084)	−0.081 (−0.242, 0.079)	−0.061 (−0.198, 0.077)
Model 2	−0.108 (−0.224, 0.009)	−0.250 (−0.537, 0.037)	−0.057 (−0.190, 0.076)	−0.084 (−0.250, 0.082)	−0.094 (−0.234, 0.047)
**ARV-USe**					
Model 1	−0.001 (−0.003, 0.001)	0.000 (−0.005, 0.005)	−0.000 (−0.003, 0.002)	−0.002 (−0.004, 0.001)	0.000 (−0.002, 0.003)
Model 2	−0.002 (−0.005, 0.000)	−0.004 (−0.010, 0.003)	−0.001 (−0.004, 0.002)	−0.002 (−0.006, 0.001)	−0.002 (−0.005, 0.001)
**VIM-USe**					
Model 1	−0.005 (−0.010, 0.001)	−0.010 (−0.024, 0.003)	−0.003 (−0.009, 0.003)	−0.003 (−0.011, 0.004)	−0.005 (−0.011, 0.002)
Model 2	−0.005 (−0.010, 0.001)	−0.010 (−0.024, 0.003)	−0.003 (−0.009, 0.003)	−0.003 (−0.011, 0.004)	−0.005 (−0.011, 0.002)
	**Uric acid**	**SBP**	**DBP**	**MAP**	**Waistline**
**Mean-USe**					
Model 1	0.000 (−0.003, 0.004)	−0.024 (−0.246, 0.197)	−0.050 (−0.164, 0.065)	−0.036 (−0.172, 0.101)	**−0.105 (−0.195, −0.010)**
Model 2					
+SD-USe	−0.001 (−0.006, 0.004)	−0.005 (−0.352, 0.341)	−0.094 (−0.276, 0.088)	−0.072 (−0.287, 0.143)	−0.128 (−0.271, 0.014)
+CV-USe	−0.002 (−0.005, 0.002)	−0.005 (−0.252, 0.241)	−0.050 (−0.180, 0.080)	−0.035 (−0.188, 0.119)	**−0.135 (−0.234, −0.031)**
+ARV-USe	−0.002 (−0.007, 0.002)	−0.083 (−0.399, 0.232)	−0.138 (−0.303, 0.027)	−0.125 (−0.320, 0.070)	**−0.149 (−0.278, −0.019)**
+VIM-USe	−0.002 (−0.005, 0.002)	−0.029 (−0.267, 0.208)	−0.050 (−0.175, 0.076)	−0.040 (−0.188, 0.109)	**−0.133 (−0.228, −0.033)**
**SD-USe**					
Model 1	−0.001 (−0.004, 0.001)	−0.029 (−0.204, 0.148)	−0.005 (−0.098, 0.088)	−0.006 (−0.115, 0.104)	−0.074 (−0.144, 0.002)
Model 2	−0.001 (−0.004, 0.003)	−0.026 (−0.281, 0.230)	0.045 (−0.089, 0.180)	0.032 (−0.126, 0.192)	−0.005 (−0.107, 0.103)
**CV-USe**					
Model 1	−0.006 (−0.126, 0.115)	−3.498 (−11.924, 4.930)	−0.389 (−4.838, 4.056)	−1.080 (−6.324, 4.171)	−0.984 (−4.481, 2.563)
Model 2	0.009 (−0.114, 0.134)	−3.451 (−12.177, 5.278)	0.076 (−4.529, 4.673)	−0.763 (−6.194, 4.671)	0.283 (−3.302, 3.891)
**ARV-USe**					
Model 1	−0.000 (−0.003, 0.002)	0.016 (−0.133, 0.166)	0.028 (−0.051, 0.106)	0.030 (−0.062, 0.123)	−0.045 (−0.106, 0.018)
Model 2	0.000 (−0.002, 0.003)	0.050 (−0.147, 0.248)	0.085 (−0.019, 0.188)	0.081 (−0.040, 0.204)	0.016 (−0.064, 0.098)
**VIM-USe**					
Model 1	0.000 (−0.005, 0.006)	−0.192 (−0.594, 0.210)	−0.020 (−0.233, 0.192)	−0.062 (−0.313, 0.188)	0.018 (−0.151, 0.187)
Model 2	0.000 (−0.005, 0.006)	−0.192 (−0.593, 0.211)	−0.019 (−0.232, 0.192)	−0.062 (−0.312, 0.189)	0.021 (−0.146, 0.187)
	**Hipline**	**Waist-hip ratio**	**MSS_sex**		
**Mean-USe**					
Model 1	−0.075 (−0.147, 0.001)	−0.000 (−0.001, 0.000)	0.009 (−0.028, 0.047)		
Model 2					
+SD-USe	−0.073 (−0.186, 0.041)	−0.001 (−0.002, 0.000)	0.017 (−0.042, 0.077)		
+CV-USe	**−0.085 (−0.164, −0.003)**	−0.001 (−0.001, 0.000)	0.002 (−0.040, 0.045)		
+ARV-USe	−0.085 (−0.186, 0.019)	−0.001 (−0.002, 0.000)	0.000 (−0.053, 0.055)		
+VIM-USe	**−0.085 (−0.161, −0.005)**	−0.001 (−0.001, 0.000)	−0.001 (−0.041, 0.041)		
**SD-USe**					
Model 1	−0.051 (−0.107, 0.008)	−0.000 (−0.001, 0.000)	−0.009 (−0.039, 0.021)		
Model 2	−0.012 (−0.094, 0.073)	0.000 (−0.001, 0.001)	−0.019 (−0.062, 0.026)		
**CV-USe**					
Model 1	−0.768 (−3.527, 2.019)	−0.003 (−0.029, 0.023)	−0.410 (−1.852, 1.040)		
Model 2	0.024 (−2.827, 2.876)	0.003 (−0.024, 0.030)	−0.433 (−1.929, 1.064)		
**ARV-USe**					
Model 1	−0.035 (−0.083, 0.015)	−0.000 (−0.001, 0.000)	−0.001 (−0.026, 0.025)		
Model 2	−0.000 (−0.064, 0.064)	0.000 (−0.000, 0.001)	−0.001 (−0.035, 0.033)		
**VIM-USe**					
Model 1	0.006 (−0.128, 0.138)	0.000 (−0.001, 0.001)	−0.019 (−0.089, 0.050)		
Model 2	0.007 (−0.125, 0.139)	0.000 (−0.001, 0.001)	−0.019 (−0.089, 0.050)		

*Boldface type indicates effect estimates were statistically significant, p-values < 0.05*.

## Discussion

### Interpretation of Results

#### Associations of Selenium Biomarkers Concentrations With Cardiometabolic Risk Factors

In this three-wave repeated-measures study of older adults with high selenium levels, we discovered that whole blood selenium was positively associated with total cholesterol, LDL-C, glucose, and uric acid. Urinary selenium was not associated with any cardiometabolic risk factors except for BP among older adults with high selenium levels.

According to epidemiological studies conducted in China and other countries, the participants in our study had high selenium, with a geometric mean (geometric SD) whole blood selenium level of 134.30 (1.19) μg/L. Although this figure is lower than that recorded in the Enshi area, which is well-known for selenium poisoning in China (66), it exceeds the median level obtained in Wuhan (92.66 μg/L) (12) and mean level observed in Shandong (120 μg/L) (24). In addition, our study population had a lower selenium level than the mean of NHANES respondents in the United States (2015–2016: 191 μg/L) (16) but a higher level than that obtained in the United Kingdom (mean: 86.856 μg/L) (67). The high selenium of our participants may have two explanations: (1) As mentioned, the study settings have selenium-rich soil. (2) Our participants were older adults and they were reported to preferred cereals rich in selenium (68). The low rates of alcohol and cigarette consumption and metabolic and physiological function (e.g., excretion) impairment that occurs during the aging process may also have contributed to the participants' high selenium levels (69, 70).

Consistent with our study, a cross-sectional study of 8,198 Chinese participants from rural areas discovered that serum selenium (mean: 120 μg/L) was positively correlated with triglycerides (0.24, 95% CI: 0.17, 0.31), total cholesterol (0.57, 95% CI: 0.49, 0.65), LDL-C (0.37, 95% CI: 0.32, 0.42), and HDL-C (0.12, 95% CI: 0.08, 0.16) (24). A cross-sectional analysis using NHANES data (2011–2012, N = 2,287) revealed that serum selenium (mean: 192.99 μg/L) was positively associated with LDL-C (0.063, 95% CI: 0.016, 0.110) (5). However, a cross-sectional analysis using NHANES data (2007–2014, *N* = 7,597) revealed that dietary selenium intake was negatively associated with total cholesterol and HDL-C (71). Furthermore, few longitudinal studies have been conducted, and those that have been conducted have reported controversial results. A 21-year follow-up analysis of the Young Finns Study and an 8-year follow-up analysis of the Olivetti Heart Study discovered positive cross-sectional associations between selenium and lipid concentrations but no such longitudinal relations (72, 73). The inconsistency between the results of our study and those of the two longitudinal studies might be partly attributable to the different population characteristics, substantial disparity in selenium levels, and potential non-linearity of the associations of selenium with lipid profiles. First, the participants of the Young Finns Study and the Olivetti Heart Study were children (aged 3–18 years) and adult males, whose physiological characteristics differ from those of a population of older adults. Second, the mean (SD) selenium concentrations reported in those two studies were 74.3 (14.0) and 77.5 (18.4) μg/L, respectively, which differed from the 134.30 (1.19) μg/L observed in our study. Therefore, large repeated-measures studies of participants with different selenium statuses and demographic characteristics are warranted to further investigate the associations of selenium and lipid profiles and the exposure–response curves.

Whole blood selenium and glucose were positively associated in older adults with high selenium. A systematic review of 15 observational studies enrolling 32,728 participants observed a positive association between selenium concentration and the odds ratio (OR) for diabetes, with a summary OR of 2.03 (95% CI: 1.51, 2.72) (74). Specifically, a cross-sectional analysis of 8,142 middle-aged adults (mean serum selenium: 121.5 μg/L) residing in Linyi, China, revealed that, compared with a low-selenium group (<124.9 μg/L), the ORs for elevated fasting serum glucose for two high-selenium groups (124.9–143.9 and >143.9 μg/L) were 2.31 (95% CI: 1.37, 3.90) and 2.67 (95% CI: 1.59, 4.48) (75). Moreover, a cross-sectional study of NHANES data (1999–2006, *N* = 41,474) reported that serum selenium concentration (mean: 129 μg/L) was positively associated with plasma glucose (12.454, 95 % CI: 4.122, 20.786) (76). Another NHANES-based (2003–2004, *N* = 917) cross-sectional analysis of adults aged over 40 years revealed a higher OR of serum selenium–related diabetes (mean: 137.1 mg/L) in women (OR: 5.99) than in men (OR: 2.30) (9). In combination with these results, our study suggests that an individual's sex may affect the selenium–glucose association.

The associations between selenium and other cardiovascular risk factors such as uric acid have not been widely studied. A positive association between whole blood selenium and uric acid among older adults with high selenium levels was observed in the present study. Consistent with our findings, a cross-sectional study of 1,406 Han Chinese adults revealed a positive association between serum selenium and the odds of hyperuricaemia, with an OR of 1.50 (95% CI: 1.01, 2.23) (77). In addition, selenium intake exceeding the recommended amount was positively correlated with the uric acid levels in the first (mean: 53.99 μg/day) and second (mean: 58.93 μg/day) trimesters of pregnancy for 95 Polish women (78). Previous findings suggest that the relationships of selenium biomarkers with uric acid may be confounded by diet and physical activity (79). All three visits in our repeated-measures study were conducted in winter to minimize the effect of seasonally relevant confounding factors, including diet and physical activity which could vary by seasons. High uric acid has been identified as an independent risk factor for long-term CVD events, CVD-related death, and all-cause mortality (80, 81). Furthermore, the positive relationship between selenium and uric acid observed in this study should be interpreted with caution, and additional comprehensive studies are required.

Few studies have explored the relationship between urinary selenium and BP. However, in the present study, we observed that urinary selenium as a continuous variable was negatively associated with SBP, DBP, and MAP among older adults with high selenium levels; however, these negative associations were non-significant when urinary selenium was treated as a categorical variable. A cross-sectional study conducted in Wuhan, China (*N* = 823), revealed a positive association between increased ORs for hypertension and urinary selenium quartiles (geometric mean: 19.8 μg/g creatinine) by using a single-metal regression model (*p* trend < 0.05) (26). However, another case–control study conducted in Wuhan (*N* = 1,004) reported that urinary selenium (geometric mean: 20.47 μg/g creatinine) was not associated with hypertension (82). This disparity can be partly attributed to the different study design and the fact that urinary selenium accounts for the majority of absorbed selenium that is not retained and is easily influenced by diet and physical activity (83).

#### Associations of VVV and IM of Selenium Biomarkers With Cardiometabolic Risk Factors

In this three-wave repeated-measures study of older adults with high selenium status, the reproducibility of the whole blood selenium measurements was high, and that of urinary selenium was poor. Because the collection of blood samples is invasive, the reproducibility of whole blood selenium measurement has rarely been studied. A longitudinal analysis of urine samples collected from 11 Chinese men at days 0, 1, 2, 3, 4, 30, 60, and 90 suggested poor reproducibility of urinary selenium (ICC = 0.03) (84). The reproducibility of whole blood and urinary selenium measurements may be affected by the kinetics of selenium absorption, distribution, metabolism, and elimination (85). Selenium is absorbed and accumulates in the human body, mainly in the liver, kidneys, and blood. The elimination of selenium in urine is also influenced by diet, lifestyle, and activity (83). Therefore, the reproducibility of whole blood selenium measurement is superior to that of urinary selenium. The reproducibility of the selenium biomarkers observed in the present study suggests that single measurements may not accurately reflect individual exposure to selenium over time, at least among older adults with high selenium, and repeated-measures studies with even more waves should be employed to minimize exposure misclassification.

We explored the relations of the VVV and IM of selenium biomarkers with cardiometabolic risk factors in the present study. Among the older adults with high selenium levels, the VVV of the selenium biomarkers was not significantly associated with cardiometabolic risk factors after adjustments for IM. The VVV of whole blood selenium, including SD-WBSe and ARV-WBSe, was associated with cardiometabolic factors, including glucose, triglycerides, and MSS-sex, but these associations were non-significant after adjustment for IM. VIM-WBSe was also not associated with any cardiometabolic risk factors. Four indicators were calculated to comprehensively evaluate the VVV of the selenium biomarkers. However, in whole blood selenium, these four indicators of VVV were not all associated with any particular cardiometabolic risk factors. Moreover, no stable association between the VVV of urinary selenium and any cardiometabolic risk factor was discovered. Although we did not observe associations between the VVV of the selenium biomarkers and cardiometabolic risk factors among older adults with high selenium levels, future research is suggested. This three-wave repeated-measures study was conducted from November 2016 through January 2018 and captured the long-term trends of the selenium biomarkers. However, repeated-measures studies conducted in different seasons are warranted to characterize seasonal biomarker variability. The VVV of the selenium biomarkers remains an informative measure for selenium biomarkers, and the potential relations of this measure with cardiometabolic risk factors should not be ignored.

We also observed that the IM of whole blood selenium was positively correlated with cardiometabolic factors, including total cholesterol, triglycerides, LDL-C, and MSS-sex, and these associations remained significant after adjustment for VVV. The IM of urinary selenium was also significantly associated with cardiometabolic risk factors after adjustment for VVV. Specifically, the IM of urinary selenium was positively related with triglycerides and glucose and negatively associated with waistline and hipline. In short, the IM of selenium biomarkers can be used in conjunction with the absolute levels for prediction of cardiometabolic risk factors among older adults with high selenium levels. The IM of the selenium biomarkers reflected the centralized levels across the study period. In sum, cardiometabolic risk is associated with exposure to selenium over time among older adults with high selenium levels. Furthermore, a pooled analysis of two prospective population-based cohort studies (i.e., the Health Retirement Study and the English Longitudinal Study of Aging, *n* = 6,237) revealed that a 1% increase in mean glycosylated hemoglobin A1c was associated with a faster rate of memory function decline (−0.041, 95% CI: −0.071, −0.012) (40). These and our findings suggest that IM in biomarkers and physiological parameters may have an impact on health end points and other studies exploring the relationship between nutrition and disease should consider the impact of IM.

In conclusion, the associations between whole blood and urinary selenium and cardiometabolic risk factors are inconsistent. Except for VVV, concentrations and IM of whole blood selenium were associated with cardiometabolic risk factors. In contrast, only VVV of urinary selenium was significantly associated with cardiometabolic risk factors. These differences could be explained for several reasons. First, selenium enters into the human body mainly by three routes (i.e., digestion, inhalation and skin absorption) (86). Whole body retention studies following oral administration of sodium selenite have indicated that human bodies absorb and accumulate selenium, primarily in the liver, kidneys, and blood (half-life: almost 9 months). The urinary excretion of selenium lasted ~1 week, with a half-life of 8–9 days (87). Hence, whole blood selenium reflected internal burden of selenium and urinary selenium reflected absorbed but unutilized selenium. This selenium metabolism may partly explain the differences in the associations observed in urinary and whole blood selenium. Second, the elimination of urinary selenium is also influenced by diet, lifestyle, and activity (83). Our data also found that the reproducibility of urinary selenium measurement was lower to that of whole blood selenium. Tough we observed negative associations of urinary selenium and SBP, DBP, and MAP, these associations were non-significant when urinary selenium concentration was treated as a categorical variable. Finally, single measurements may not accurately reflect selenium status. IM can capture the centralizing trend of multiple repeated measurements. We did not observe negative association between IM of urinary selenium and BP-related factors. However, both whole blood selenium concentration and IM of whole blood selenium were positively associated with total cholesterol, LDL-C, glucose, and uric acid. Hence, association of whole blood selenium are more stable than that of urinary selenium. Even though the available evidence can partially explain the inconsistent results in the associations observed in urinary and whole blood selenium, experimental animal studies exploring the underlying mechanisms are warrant.

As an essential trace element, selenium is incorporated into selenoproteins that have a wide range of dose-dependent effects. The *U*-shaped associations between selenium levels and cardiometabolic risk factors must be emphasized (10). A case–control study conducted in Wuhan demonstrated a *U*-shaped association between plasma selenium (median: 92.66 μg/L) with central obesity and high blood pressure (12). As above mentioned, another cross-sectional analysis using data from NHANES reported a *U*-shaped association between plasma selenium and the likelihood of diabetes, with the lowest risk noted for a concentration of ~122 μg/L (9). Due to the high blood selenium status (GM:134.30 μg/L), the positive association between whole blood selenium and blood glucose was observed in our study. Additionally, the *U*-shaped association was observed between selenium status and all-cause and cancer mortality among 13,887 adult participants in the NHANES (1988-1994), with the lowest risk noted for a concentration of ~135 μg/L (88). In order to analyse the two-way effect of selenium on health, especially cardiometabolic risk factors, more epidemiological studies of participants with wide selenium concentration statuses and demographic characteristics were warranted. The exposure response curve of selenium biomarkers and cardiometabolic risk factors among both deficient and excessive selenium levels were needed to be profiled.

### Potential Mechanisms

The biological mechanisms explaining selenium's involvement in the pathogenesis of cardiometabolic diseases are not well-understood. High levels of selenium are incorporated into selenoproteins, damaging cardiometabolic health mainly by initiating oxidative stress, promoting insulin resistance, and regulating gluconeogenesis and lipid metabolism. Excess selenium increases the production of reactive oxygen species (ROS) by promoting the overexpression of selenocysteine transfer RNA and reducing selenoproteins synthesis. Increased ROS levels cause insulin resistance in the peripheral tissues by affecting insulin receptor signal transduction, ultimately resulting in hyperinsulinemia and cell glucose desensitization (89). In an experimental study, C57BL/6J mice (*n* = 6 or 7 per group) on a selenium-supplemented diet (0.1 and 0.4 ppm selenium) developed hyperinsulinemia and reduced insulin sensitivity (90). In addition, excessive ROS production can reduce nitric oxide; accelerate endothelial cell apoptosis; induce upregulation of NF-kB; activate intercellular adhesion molecule-1, monocyte chemotactic protein-1, and vascular cell adhesion molecule-1; and trigger diabetic vascular complications and cardiometabolic disorders (91).

Meanwhile, high dietary selenium intake can increase the expression or activity of key proteins related to gluconeogenesis, glycolysis, and lipogenesis by upregulating the selenoproteins glutathione peroxidase family. First, high glutathione peroxidase-1 production due to high selenium intake results in the upregulation of phosphoenolpyruvate carboxykinase and upregulation of fatty acid synthesis. In an experimental study, prolonged high dietary intake of selenium was demonstrated to induce gestational diabetes in rats and hyperinsulinemia in pigs (92). Second, high selenium upregulates protein tyrosine phosphatase 1B (PTP1B), a key enzyme in triggering fatty acid synthesis and in reverse regulation of insulin signaling, eventually inducing lipid disorders and insulin resistance. In one animal study, PTP1B expression were elevated in rats with fructose-rich diets, resulting in the induction of fatty acid synthesis and an increase in liver triglycerides (93). Further studies are warranted to clarify the mechanisms underlying the complex associations between selenium and cardiometabolic risk.

### Limitations and Strengths

This study had several limitations. First, we selected whole blood selenium and urinary selenium as the selenium biomarkers to investigate, but serum or plasma selenium may have been more favorable alternatives. Nevertheless, in our study, whole blood selenium and urinary selenium were preferable because of the consistency of concentrations and determinants between the whole blood and serum selenium; in addition, whole blood selenium is reportedly a suitable indicator of medium- to long-term selenium status (94). Moreover, measuring both whole blood and urinary selenium provides an accurate picture of the function and excretion characteristics of selenium (83). Second, information regarding the participants' alcohol consumption, cigarette smoking habits, and disease history was collected using questionnaires. Consequently, unmeasured confounding factors and recall bias likely limit the generalisability of our findings. Third, the modest number of repeat measurements and their short time intervals might have hampered our ability to characterize selenium variability. Despite the limited data, we provide a fresh perspective on the relationships between selenium and cardiometabolic risk factors. Finally, the participants in our study were older adults (age ≥50 years) with high selenium levels; thus, the generalisability of the results to more general populations may be limited.

Despite these limitations, several strengths should also be considered. The three-wave repeated-measures design enabled repeated data collection and inference of causal relationships. Additionally, all three measurements were conducted in winter, minimizing the effect of seasonally relevant confounding factors. To our knowledge, this is the first study to comprehensively evaluate the associations of selenium concentration, VVV, IM with cardiometabolic risk factors. Finally, our findings are derived from observations of older adults with high blood selenium in the Beijing region, elucidating the health effects of high selenium and providing science-based evidence for nutritional guidelines in the region.

## Conclusion

We discovered that selenium levels and their IMs were significantly associated with several cardiometabolic factors, namely total cholesterol, LDL-C, and glucose, in older adults with high selenium in the Beijing area. This indicates that selenium affects cardiometabolic risk. However, we do not observe any robust associations between the VVV of the selenium biomarkers and cardiometabolic risk factors after adjustment for IM. The findings suggest that older adults with high selenium should not take dietary selenium supplements to prevent cardiometabolic risk. In the future, longer repeated-measures studies of the general population are warranted to minimize selenium exposure misclassification, explore the associations of the VVV and IM of selenium biomarkers with cardiometabolic risk factors, and determine the relevant underlying mechanisms.

## Data Availability Statement

The datasets presented in this study can be found in online repositories. The names of the repository/repositories and accession number(s) can be found in the article/Supplementary Material.

## Author Contributions

AL: conceptualization, methodology, data-analysis and interpretation, writing-original draft, writing-review and editing, and funding acquisition. QZ: data-cleaning and interpretation, writing-original draft, and writing-review and editing. YM and JZ: investigation, data cleaning, and writing-review and editing. MZ, JX, and XG: investigation and writing-review and editing. QX: funding acquisition, writing-review and editing, and supervision. All authors contributed to the article and approved the submitted version.

## Funding

This study was supported by the China Medical Board (Grant No. 15-230), the Fundamental Research Funds for the Central Universities (Grant No. 3332019147), Peking Union Medical College Graduate Innovation Fund (No. 2019-1004-02), the China Prospective cohort study of Air pollution and health effects in Typical areas (C-PAT; Grant No. MEE-EH-20190802), and the Chinese Academy of Medical Science Innovation Fund for Medical Sciences (Grant No. 2017-I2M-1-009).

## Conflict of Interest

The authors declare that the research was conducted in the absence of any commercial or financial relationships that could be construed as a potential conflict of interest.

## Publisher's Note

All claims expressed in this article are solely those of the authors and do not necessarily represent those of their affiliated organizations, or those of the publisher, the editors and the reviewers. Any product that may be evaluated in this article, or claim that may be made by its manufacturer, is not guaranteed or endorsed by the publisher.

## Glossary

ARV, average real variability; ARV-Use, average real variability of urinary selenium; ARV-WBSe, average real variability of whole blood selenium; BMI, body mass index; CI, confidence interval; CV, coefficient of variation; CVD, cardiovascular disease; CV-Use, coefficient of variation of urinary selenium; CV-WBSe, coefficient of variation of whole blood selenium; DBP, diastolic blood pressure; EDF, estimated degrees of freedom; FAS, fatty acid synthesis; FDR, false discovery rate; FFQ, food frequency questionnaire; GAMMs, generalized additive mixed models; HDL-C, high-density lipoprotein cholesterol; hs-CRP, high-sensitivity C-reactive protein; ICCs, intraclass correlation coefficients; ICP-MS, inductively coupled plasma mass spectrometer; LDL-C, low-density lipoprotein cholesterol; LOD, limit of detection; MAP, mean arterial pressure; MSS, metabolic syndrome score; MSS-sex, sex-specific metabolic syndrome score; NHANES, the National Health and Nutrition Examination Survey; OR, odds ratio; PTP1B, protein tyrosine phosphatase 1B; ROS, reactive oxygen species; SBP, systolic blood pressure; SD, standard deviation; SD-USe, standard deviation of urinary selenium; SD-WBSe, standard deviation of whole blood selenium; Use, urinary selenium; VIM, variability independent of the mean; VIM-USe, variability independent of the mean of urinary selenium; VIM-WBSe, variability independent of the mean of whole blood selenium; WBSe, whole blood selenium.
