# Supplementary material for: Novel Strategies for Assessing Associations Between Selenium Biomarkers and Cardiometabolic Risk Factors: Concentration, Visit-to-Visit Variability, or Individual Mean? Evidence From a Repeated-Measures Study of Older Adults With High Selenium
Source: Front Nutr. 2022 May 30;9:838613. doi: 10.3389/fnut.2022.838613 (PMC9196882; doi:10.3389/fnut.2022.838613)
Supplement: Supplementary file 1 [file Data_Sheet_1.docx]

**Supplementary Materials**

Catalogue

[**Figure S1. Directed acyclic graph showing the relationship between selenium biomarkers (represented by the green ellipse with the triangle inside) and cardiovascular risk factors (represented by the blue ellipse with the line inside).** 2](#_Toc103698692)

[**Figure S2. Spearman correlation coefficients between each pair of selenium biomarkers across three visits.** 3](#_Toc103698693)

[**Figure S3. Distribution of selenium biomarkers concentrations across three visits.** 4](#_Toc103698694)

[**Table S1. Comparison among measured values of selenium biomarkers and cardiometabolic risk factors across three visits (measure of 1^st^ visit was severed as a reference).** 5](#_Toc103698695)

[**Table S2. Comparison among measured values of selenium biomarkers and cardiometabolic risk factors across three visits (measure of 3^rd^ visit was severed as a reference).** 6](#_Toc103698696)

[**Table S3. Distribution characteristics of selenium biomarkers by urban and rural areas.** 7](#_Toc103698697)

[**Table S4. The association between selenium biomarkers and cardiometabolic risk factors using GAMMs embedded with restricted cubic spline smoother with three degrees of freedom.** 8](#_Toc103698698)

[**Figure S4. The exposure-response relationships between whole blood selenium and cardiometabolic risk factors using GAMMs embedded with restricted cubic spline smoother with three degrees of freedom.** 9](#_Toc103698699)

[**Figure S4 - continued. The exposure-response relationships between whole blood selenium and cardiometabolic risk factors using GAMMs embedded with restricted cubic spline smoother with three degrees of freedom.** 10](#_Toc103698700)

[**Figure S5. The exposure-response relationships between urinary selenium and cardiometabolic risk factors using GAMMs embedded with restricted cubic spline smoother with three degrees of freedom.** 11](#_Toc103698701)

[**Figure S5 - continued. The exposure-response relationships between urinary selenium and cardiometabolic risk factors using GAMMs embedded with restricted cubic spline smoother with three degrees of freedom.** 12](#_Toc103698702)

[**Figure S6. Estimates and 95% confidence intervals of cardiometabolic risk factors associated with one-unit increase of natural log transformed whole blood selenium, stratified by gender, age, BMI, hypertension status, diabetes status, dyslipidaemia status.** 13](#_Toc103698703)

[**Figure S6-continued. Estimates and 95% confidence intervals of cardiometabolic risk factors associated with one-unit increase of natural log transformed whole blood selenium, stratified by gender, age, BMI, hypertension status, diabetes status, dyslipidaemia status.** 14](#_Toc103698704)

[**Figure S7. Estimates and 95% confidence intervals of cardiometabolic risk factors associated with one-unit increase of natural log transformed urinary selenium, stratified by gender, age, BMI, hypertension status, diabetes status, dyslipidaemia status.** 15](#_Toc103698705)

[**Figure S7-continued. Estimates and 95% confidence intervals of cardiometabolic risk factors associated with one-unit increase of natural log transformed urinary selenium, stratified by gender, age, BMI, hypertension status, diabetes status, dyslipidaemia status.** 16](#_Toc103698706)

[**Figure S8. Estimates and 95% confidence intervals of cardiometabolic risk factors associated with one-unit increase of natural log transformed selenium biomarkers, additionally adjusting for history of hypertension, diabetes, or dyslipidaemia on the basis of main model.** 17](#_Toc103698707)

[**Figure S9. Estimates and 95% confidence intervals of cardiometabolic risk factors associated with one-unit increase of natural log transformed selenium biomarkers, excluding BMI from covariates of main model.** 18](#_Toc103698708)

[**Figure S10. Estimates and 95% confidence intervals of cardiometabolic risk factors associated with one-unit increase of natural log transformed selenium biomarkers, additionally corrected for high‐sensitivity C‐reactive protein on the basis of main model.** 19](#_Toc103698709)


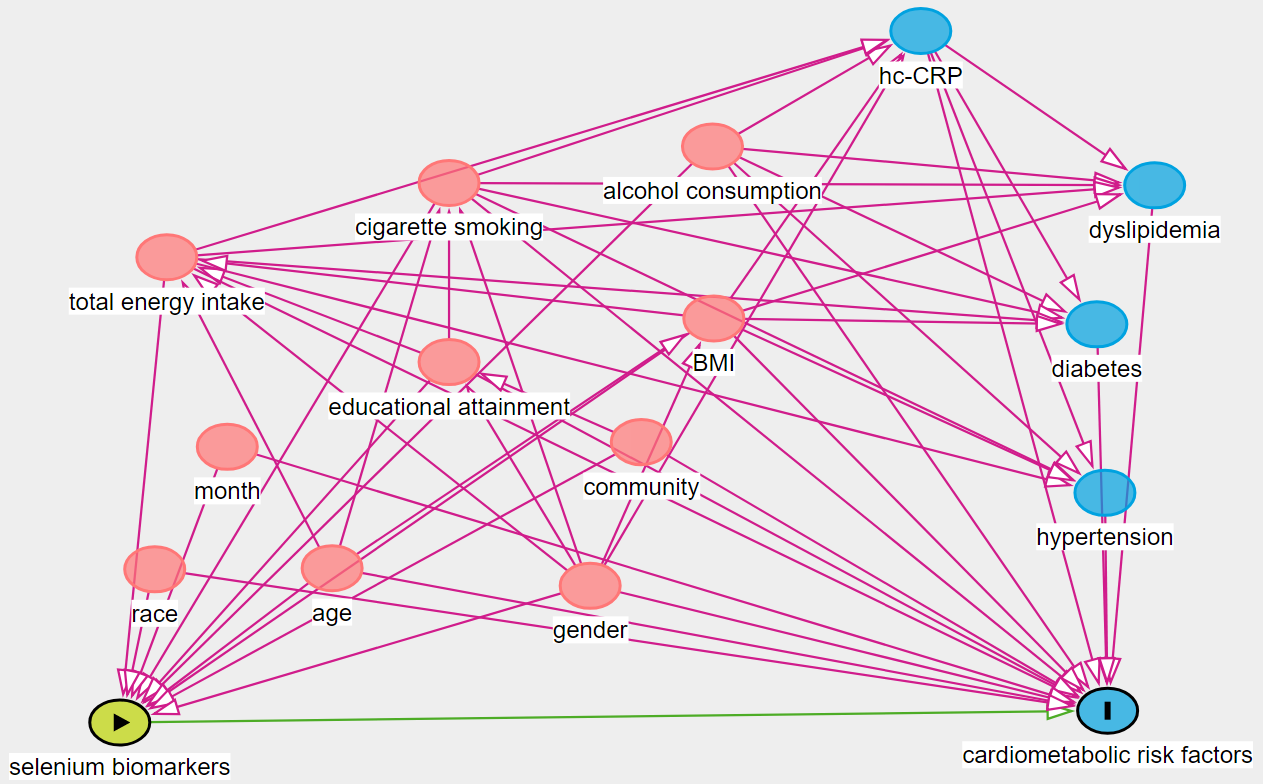


**Figure S1. Directed acyclic graph showing the relationship between selenium biomarkers (represented by the green ellipse with the triangle inside) and cardiovascular risk factors (represented by the blue ellipse with the line inside).**

In this diagram, the pink ellipse represents the ancestor of exposure and outcome. The ancestor of the outcome is represented in blue oval. Minimal sufficient adjustment sets (MSAS) for estimating the total effect of selenium biomarkers on cardiovascular risk factors included age, sex, race, BMI, educational attainment, cigarette smoking, alcohol consumption, month, community and total energy intake.

Abbreviations: BMI: Body Mass Index; hs-CRP: high‐sensitivity C‐reactive protein.


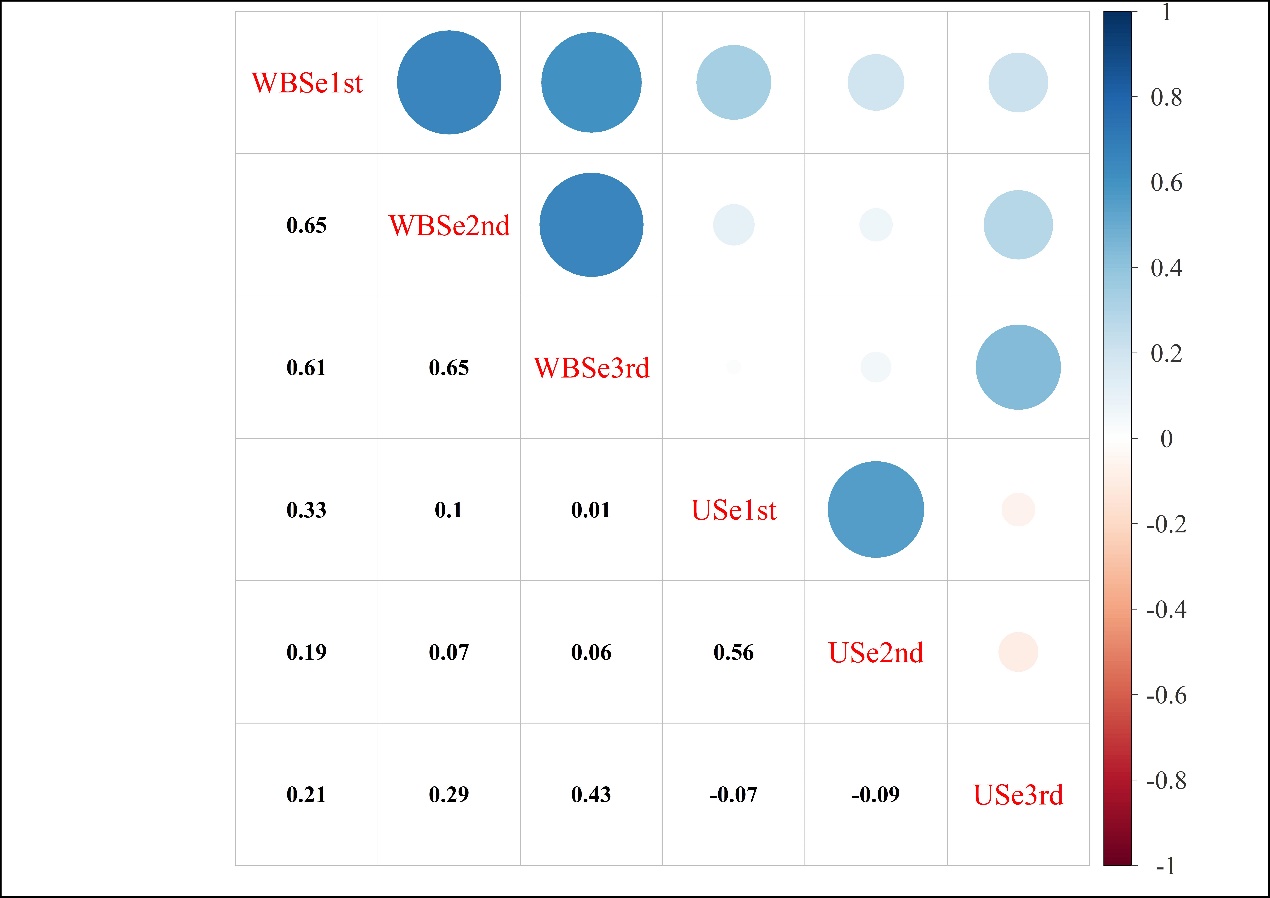


**Figure** **S2. Spearman correlation coefficients between each pair of selenium biomarkers across three visits.**

Abbreviation: WBSe1st: whole blood selenium concentrations in the first visit; WBSe2nd: whole blood selenium concentrations in the second visit; WBSe3rd: whole blood selenium concentrations in the third visit; USe1st: urinary selenium concentrations in the first visit; USe2nd: urinary selenium concentrations in the second visit; USe3rd: urinary selenium concentrations in the third visit.


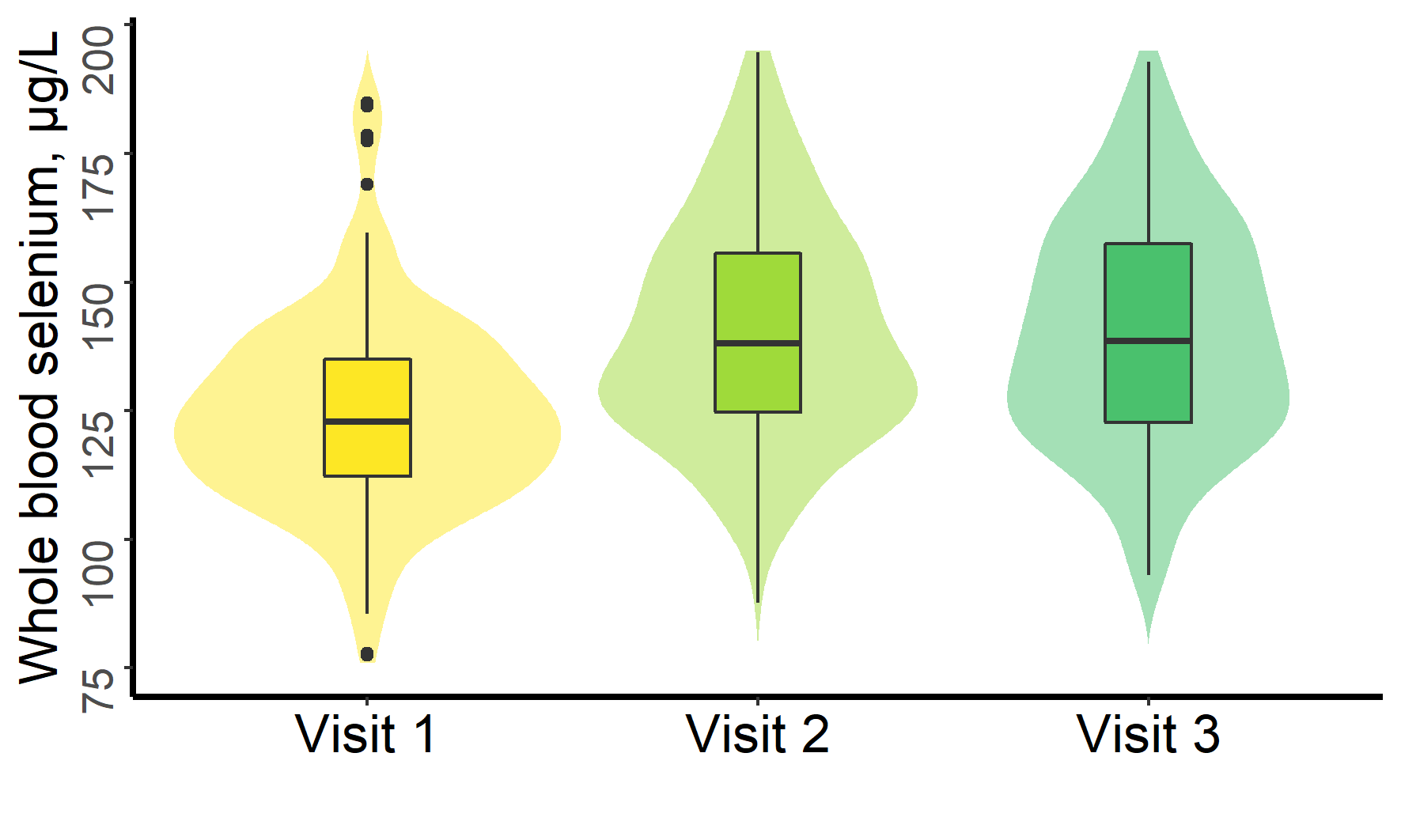


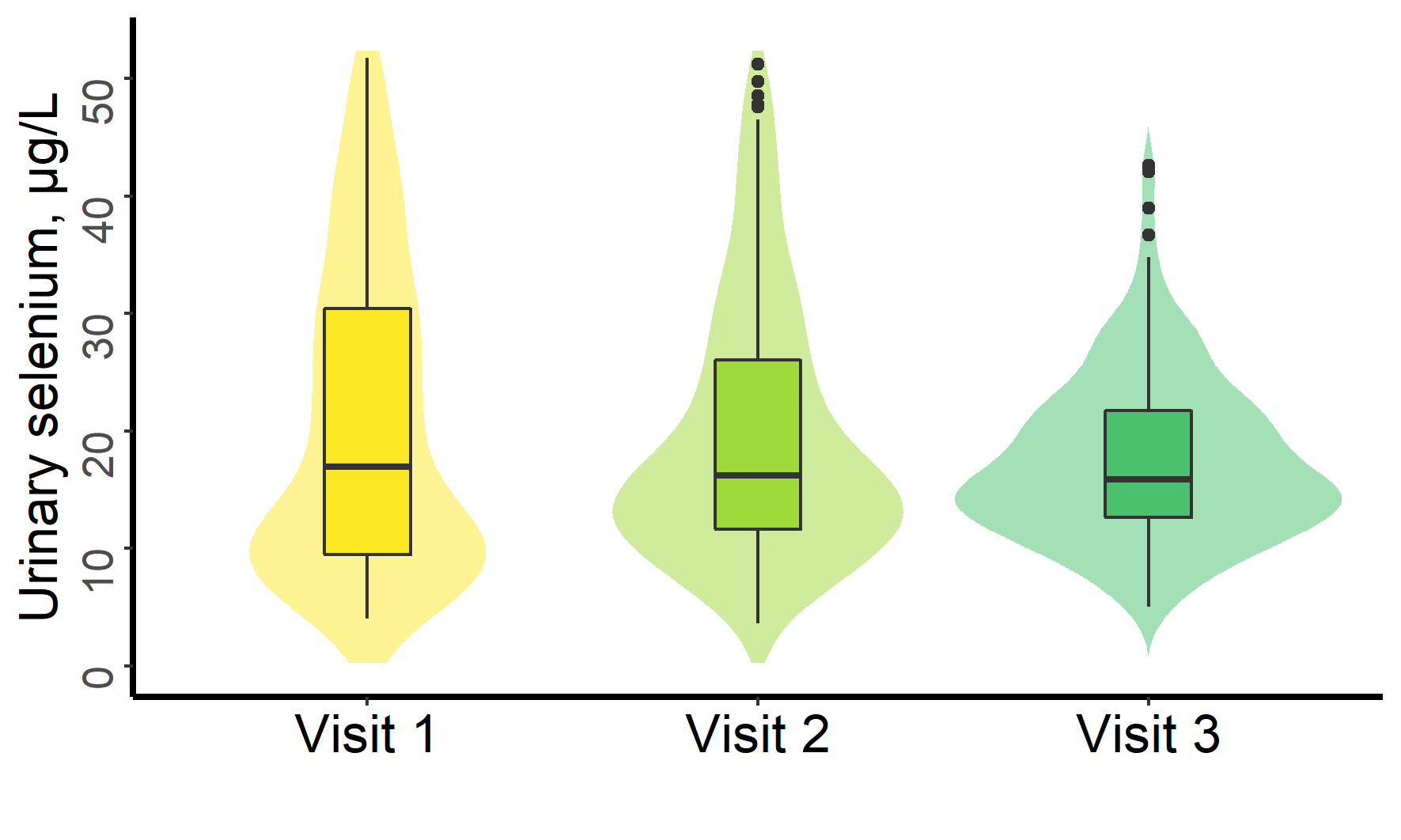


**Figure S3.** **Distribution of selenium biomarkers concentrations across three visits.**

**Table S1.** **Comparison among measured values of** **selenium biomarkers and cardiometabolic risk factors across three visits (measure of 1^st^ visit was severed as a reference).**

| *p*-values | 1^st^ Visit | 2^nd^ Visit | 3^rd^ Visit | The global  significance  test |
| --- | --- | --- | --- | --- |
| **Selenium biomarkers** | | | | |
| Whole blood selenium | ref | **<0.01** | **<0.01** | **<0.01** |
| Urinary selenium | ref | 0.59 | **0.04** | 0.09 |
|  |  |  |  |  |
| **Cardiometabolic risk factors** | | | | |
| Total cholesterol | ref | **0.04** | **<0.01** | **<0.01** |
| Triglyceride | ref | **0.02** | 0.51 | **<0.01** |
| HDL-C | ref | 0.16 | **<0.01** | **<0.01** |
| LDL-C | ref | 0.19 | **0.01** | **<0.01** |
| Glucose | ref | **<0.01** | **<0.01** | **<0.01** |
| Uric acid | ref | 0.23 | **<0.01** | **<0.01** |
| SBP | ref | 0.16 | 0.14 | 0.26 |
| DBP | ref | **<0.01** | **<0.01** | **<0.01** |
| MAP | ref | **<0.01** | **<0.01** | **<0.01** |
| Waistline | ref | **<0.01** | **0.03** | **<0.01** |
| Hipline | ref | **<0.01** | 0.76 | **<0.01** |
| Waist-hip ratio | ref | **<0.01** | 0.06 | **<0.01** |
| MSS­-sex | ref | 0.28 | **<0.01** | **<0.01** |

Abbreviations: HDL-C: high-density lipoprotein cholesterol; LDL-C: low-density lipoprotein cholesterol; SBP: systolic blood pressure; DBP: diastolic blood pressure; MAP: mean arterial pressure; MSS­-sex: metabolic syndrome score (sex specific).

Boldface type indicates comparison among measured values across visits was statistically significant (*p* < 0.05).

ref: measured values in the first visit was severed as a reference, and the second measured value and the third measured value was respectively compared with it.

**Table S2. Comparison among measured values of selenium biomarkers and cardiometabolic risk factors across three visits (measure of 3^rd^ visit was severed as a reference).**

| *p*-values | 1^st^ Visit | 2^nd^ Visit | | 3^rd^ Visit | | The global  significance  test |
| --- | --- | --- | --- | --- | --- | --- |
| **Selenium biomarkers** | | | | | | |
| Whole blood selenium | **<0.01** | 0.52 | ref | | **<0.01** | |
| Urinary selenium | **0.04** | 0.11 | ref | | 0.09 | |
|  |  |  |  | |  | |
| **Cardiometabolic risk factors** | | | | | | |
| Total cholesterol | **<0.01** | **0.03** | ref | | **<0.01** | |
| Triglyceride | 0.51 | **<0.01** | ref | | **<0.01** | |
| HDL-C | **<0.01** | **<0.01** | ref | | **<0.01** | |
| LDL-C | **0.01** | **<0.01** | ref | | **<0.01** | |
| Glucose | **<0.01** | 0.11 | ref | | **<0.01** | |
| Uric acid | **<0.01** | **<0.01** | ref | | **<0.01** | |
| SBP | 0.14 | 0.90 | ref | | 0.26 | |
| DBP | **<0.01** | 0.95 | ref | | **<0.01** | |
| MAP | **<0.01** | 0.73 | ref | | **<0.01** | |
| Waistline | **0.03** | **<0.01** | ref | | **<0.01** | |
| Hipline | 0.76 | **<0.01** | ref | | **<0.01** | |
| Waist-hip ratio | 0.06 | **0.02** | ref | | **<0.01** | |
| MSS-sex | **<0.01** | **<0.01** | ref | | **<0.01** | |

Abbreviations: HDL-C: high-density lipoprotein cholesterol; LDL-C: low-density lipoprotein cholesterol; SBP: systolic blood pressure; DBP: diastolic blood pressure; MAP: mean arterial pressure; MSS-sex: metabolic syndrome score (sex specific).

Boldface type indicates comparison among measured values across visits was statistically significant (*p* < 0.05).

ref: measured values in the third visit was severed as a reference, and the first measured value and the second measured value was respectively compared with it.

**Table S3. Distribution characteristics of selenium biomarkers by urban and rural areas.**

| Analyte (μg/L) | GM | GSD | 5^th^ | 25^th^ | 50^th^ | 75^th^ | 95^th^ | Significance test ^a^ |
| --- | --- | --- | --- | --- | --- | --- | --- | --- |
| Whole blood selenium | | | | | | | | |
| Urban area | 146.94 | 1.18 | 115.10 | 131.20 | 144.60 | 165.40 | 189.20 | *p*-values <0.001 |
| Rural area | 130.69 | 1.18 | 100.10 | 117.60 | 129.70 | 145.60 | 172.40 |  |
| Urinary selenium | | | | | | | | |
| Urban area | 22.83 | 1.98 | 7.90 | 14.50 | 22.50 | 37.50 | 72.80 | *p*-values <0.001 |
| Rural area | 16.30 | 1.77 | 7.00 | 11.00 | 15.20 | 23.60 | 44.40 |  |

Abbreviations: GM: geometric mean; GSD: geometric standard deviation.

^a^. Unadjusted linear mixed-effects regression models of the selenium biomarkers by area (urban area, rural area) were developed to assess difference of selenium biomarkers concentrations between two areas.

Among five communities in present study, Qian Nantai, Liu Hegou are located in rural area. Dongcheng, Chaoyang and Fangshan are located in urban area.

| Cardiometabolic risk factors | WBSe | |  | USe | |
| --- | --- | --- | --- | --- | --- |
|  | edf | *p*-value |  | edf | *p*-value |
| Total cholesterol | 1.22 | **p<0.01** |  | 1.17 | 0.82 |
| Triglyceride | 1.00 | 0.10 |  | 1.00 | 0.97 |
| HDL-C | 1.42 | 0.10 |  | 1.12 | 0.32 |
| LDL-C | 2.16 | **p<0.01** |  | 1.00 | 0.89 |
| Glucose | 2.14 | **p<0.01** |  | 1.00 | 0.10 |
| Uric acid | 1.53 | **0.04** |  | 1.13 | 0.68 |
| SBP | 1.00 | 0.09 |  | 1.00 | **p<0.01** |
| DBP | 1.55 | 0.33 |  | 1.00 | **0.01** |
| MAP | 1.46 | 0.77 |  | 1.00 | **p<0.01** |
| Waistline | 1.00 | 0.79 |  | 1.62 | 0.53 |
| Hipline | 1.00 | 0.91 |  | 1.00 | 0.78 |
| Waist-hip ratio | 2.22 | 0.60 |  | 1.09 | 0.92 |
| MSS-sex | 1.00 | 0.49 |  | 1.00 | **0.03** |

**Table S4.** **The association between selenium biomarkers and cardiometabolic risk factors using GAMMs embedded with restricted cubic spline smoother with three degrees of freedom.**

Abbreviations: GAMMs: generalised additive mixed models; HDL-C: high-density lipoprotein cholesterol; LDL-C: low-density lipoprotein cholesterol; SBP: systolic blood pressure; DBP: diastolic blood pressure; MAP: mean arterial pressure; MSS: metabolic syndrome score; MSS -sex: metabolic syndrome score (sex specific); WBSe: whole blood selenium; USe: urinary selenium. Boldface type indicates effect estimates were statistically significant, *p*-values < 0.05.


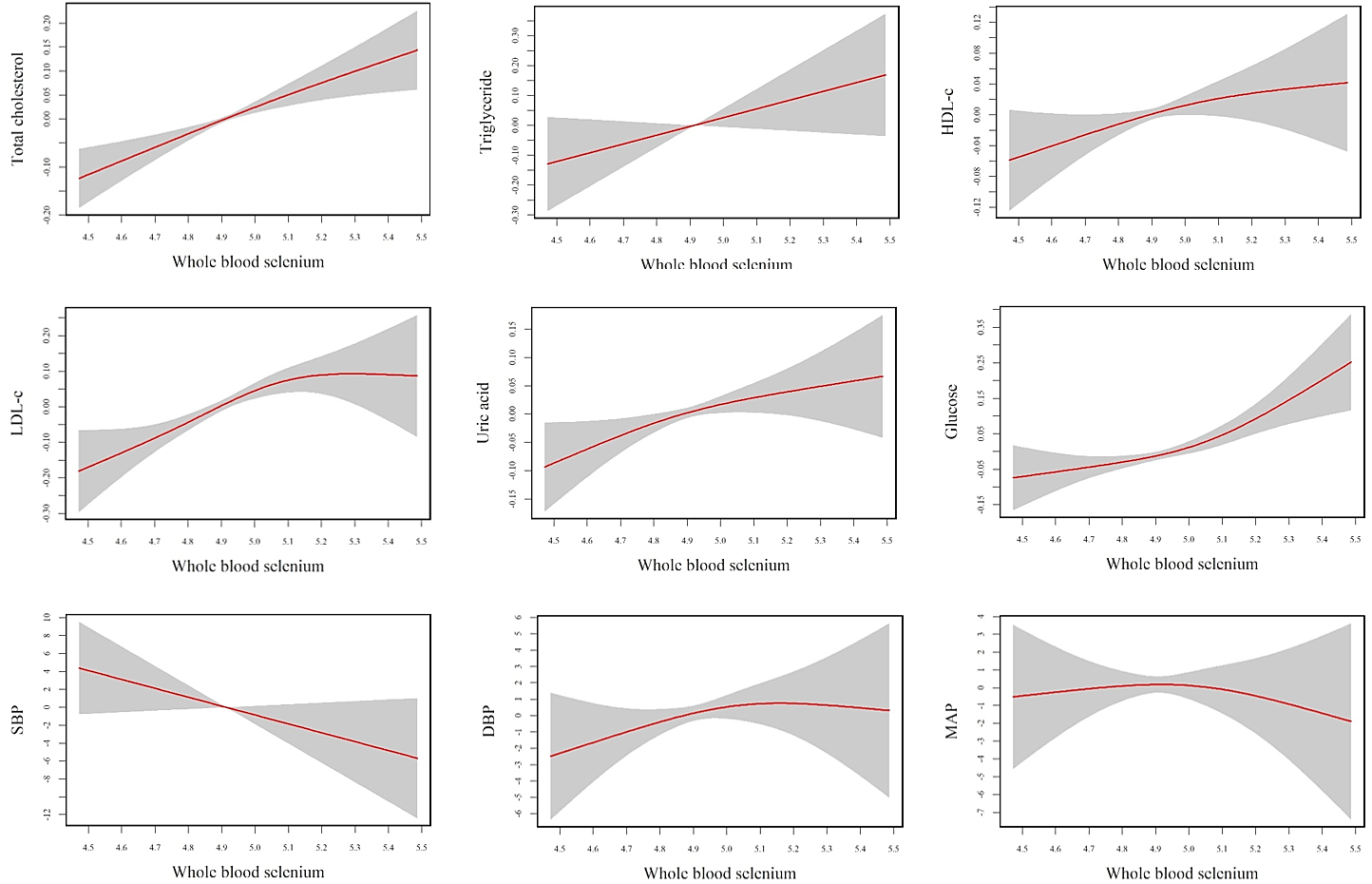


**Figure S4. The exposure-response relationships between whole blood selenium and cardiometabolic risk factors using GAMMs embedded with restricted cubic spline smoother with three degrees of freedom.**

The effect estimate is shown by the red line, and the shaded areas represent the 95% confidence intervals.

Abbreviation: GAMMs: generalized additive mixed models; HDL-C: high-density lipoprotein cholesterol; LDL-C: low-density lipoprotein cholesterol; DBP: diastolic blood pressure; SBP: systolic blood pressure. MAP: mean arterial pressure.


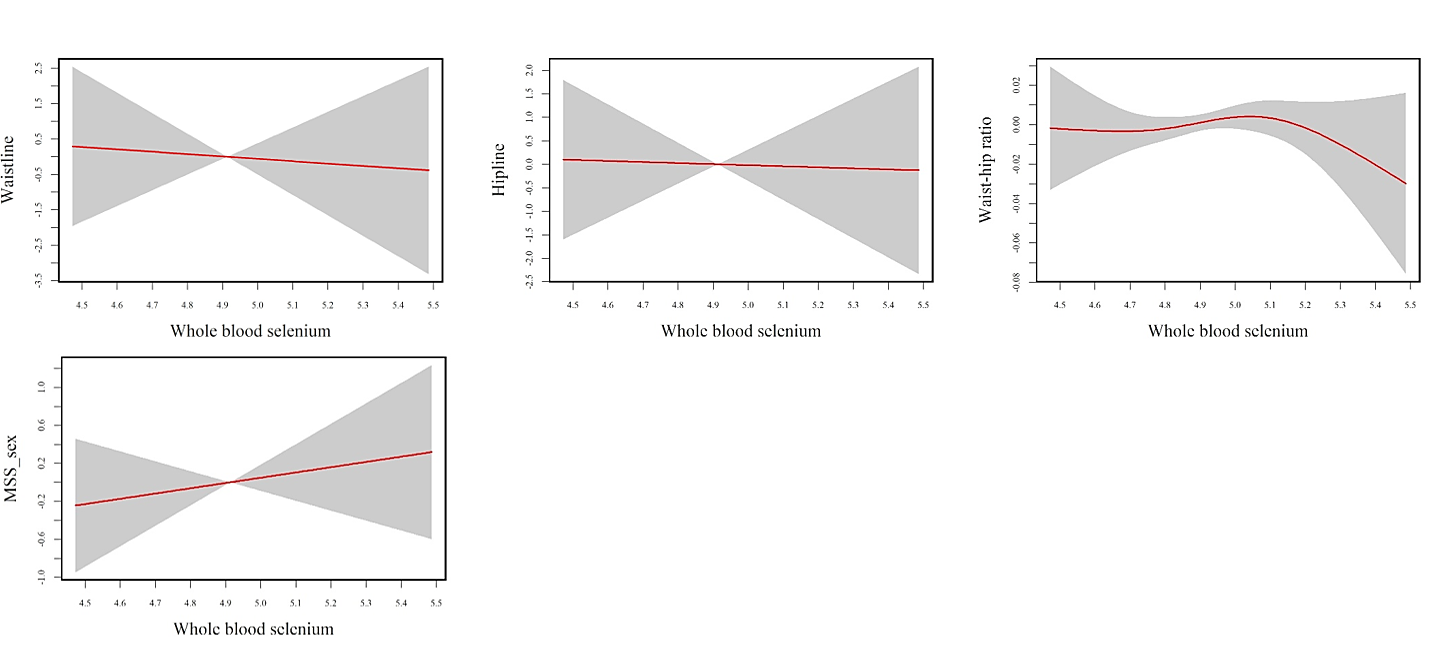


**Figure S4 - continued. The exposure-response relationships between whole blood selenium and cardiometabolic risk factors using GAMMs embedded with restricted cubic spline smoother with three degrees of freedom.**

The effect estimate is shown by the red line, and the shaded areas represent the 95% confidence intervals.

Abbreviation: GAMMs: generalized additive mixed models; MSS -sex: metabolic syndrome score (sex specific).


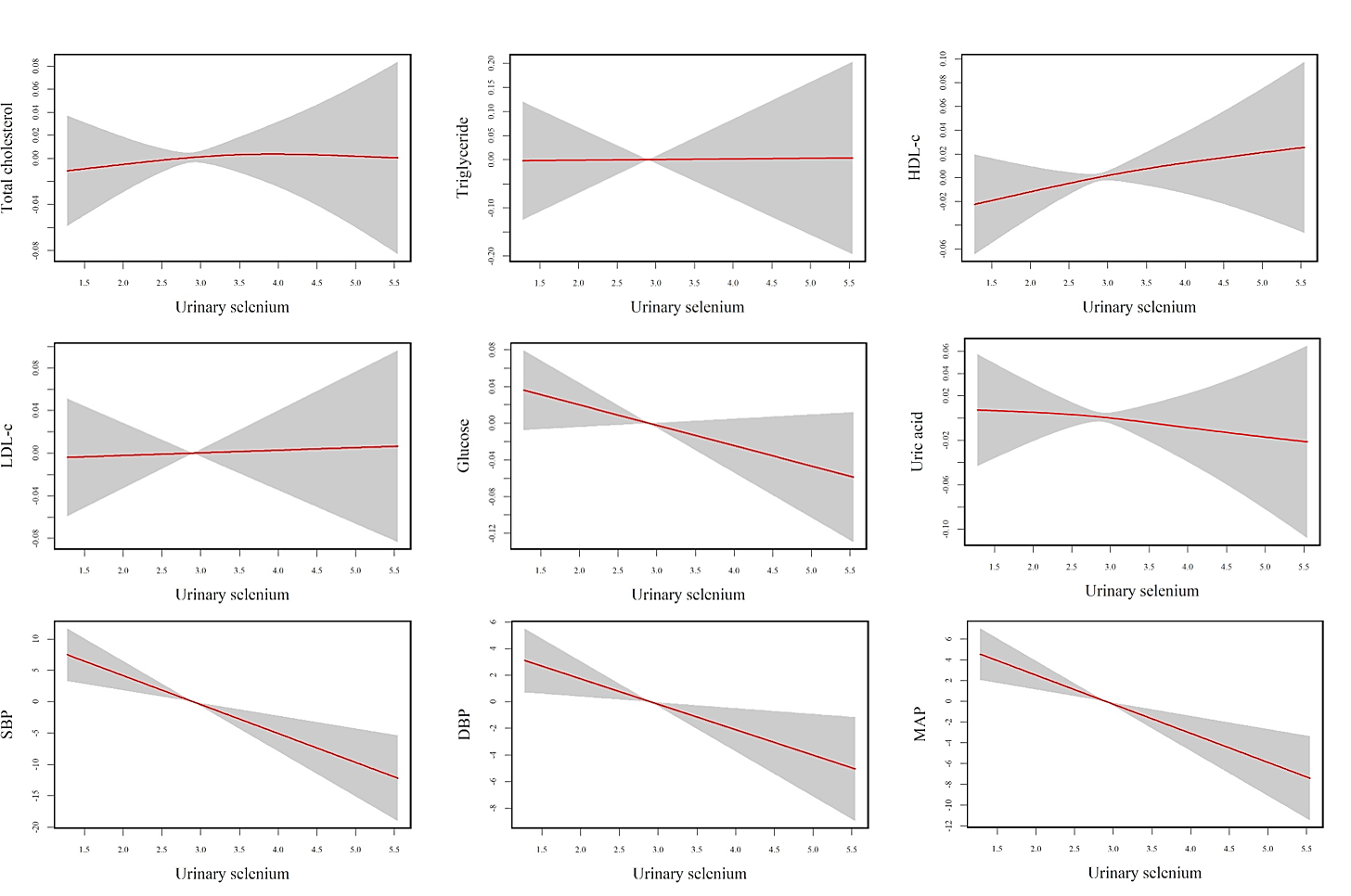


**Figure S5. The exposure-response relationships between urinary selenium and cardiometabolic risk factors using GAMMs embedded with restricted cubic spline smoother with three degrees of freedom.**

The effect estimate is shown by the red line, and the shaded areas represent the 95% confidence intervals.

Abbreviation: GAMMs: generalized additive mixed models; HDL-C: high-density lipoprotein cholesterol; LDL-C: low-density lipoprotein cholesterol; SBP: systolic blood pressure; DBP: diastolic blood pressure; MAP: mean arterial pressure.


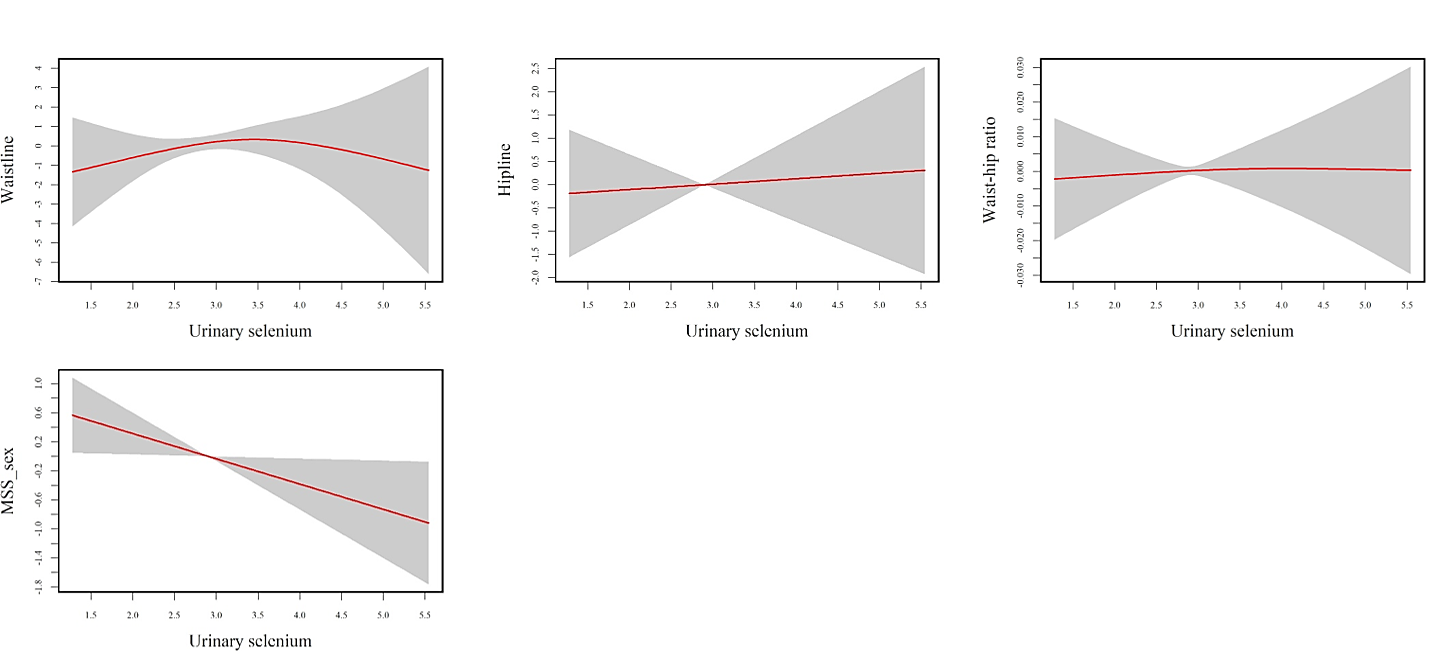


**Figure S5 - continued. The exposure-response relationships between urinary selenium and cardiometabolic risk factors using GAMMs embedded with restricted cubic spline smoother with three degrees of freedom.**

The effect estimate is shown by the red line, and the shaded areas represent the 95% confidence intervals.

Abbreviation: GAMMs: generalized additive mixed models; MSS -sex: metabolic syndrome score (sex specific).


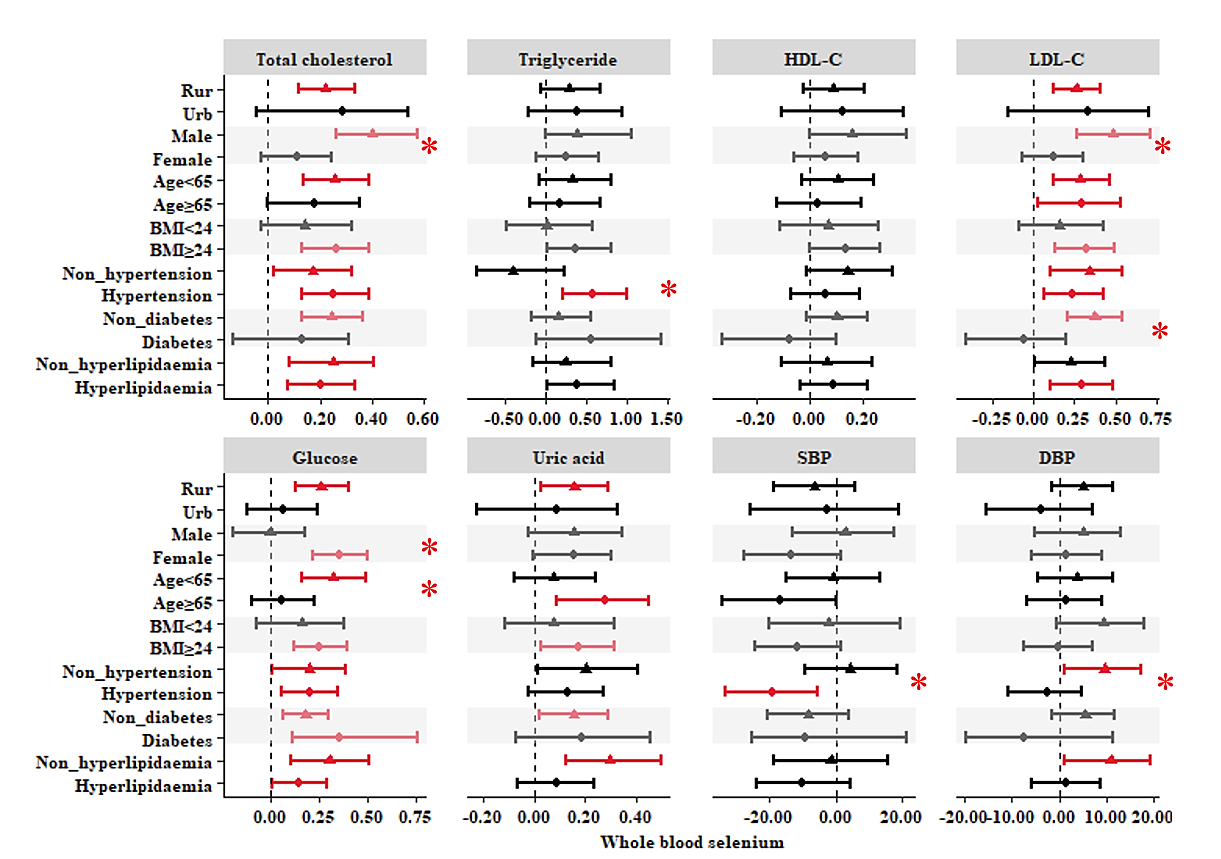


**Figure S6. Estimates and 95% confidence intervals of cardiometabolic risk factors associated with one-unit increase of natural log transformed whole blood selenium, stratified by gender, age, BMI, hypertension status, diabetes status,** **dyslipidaemia status.**

Red indicates statistical significance (*p* < 0.05). Asterisk (*) indicates significant difference in subgroups.

Abbreviation: Rur: rural area; Urb: urban area; BMI: Body Mass Index; HDL-C: high-density lipoprotein cholesterol; LDL-C: low-density lipoprotein cholesterol; SBP: systolic blood pressure; DBP: diastolic blood pressure.


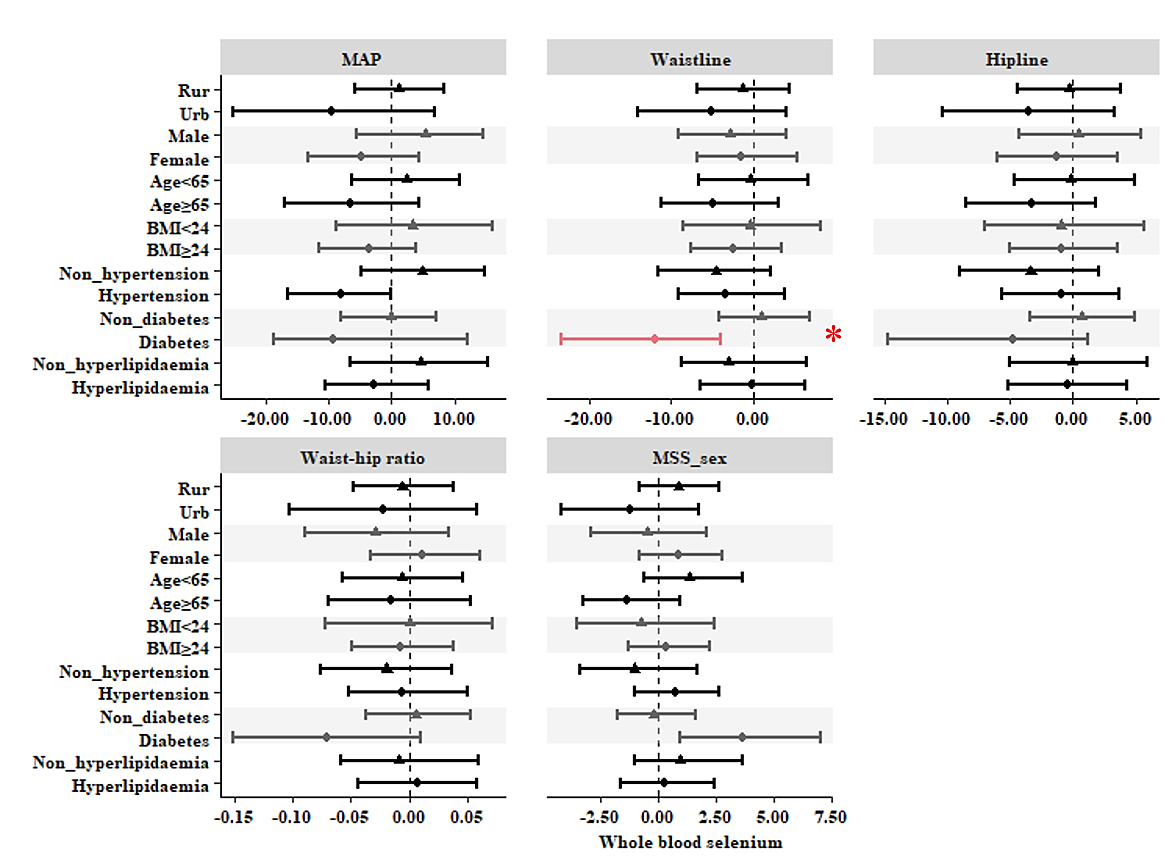


**Figure S6-continued. Estimates and 95% confidence intervals of cardiometabolic risk factors associated with one-unit increase of natural log transformed whole blood selenium, stratified by gender, age, BMI, hypertension status, diabetes status, dyslipidaemia status.**

Red indicates statistical significance (*p* < 0.05). Asterisk (*) indicates significant difference in subgroups.

Abbreviation: Rur: rural area; Urb: urban area; MAP: mean arterial pressure; MSS-sex: metabolic syndrome score (sex specific).

**
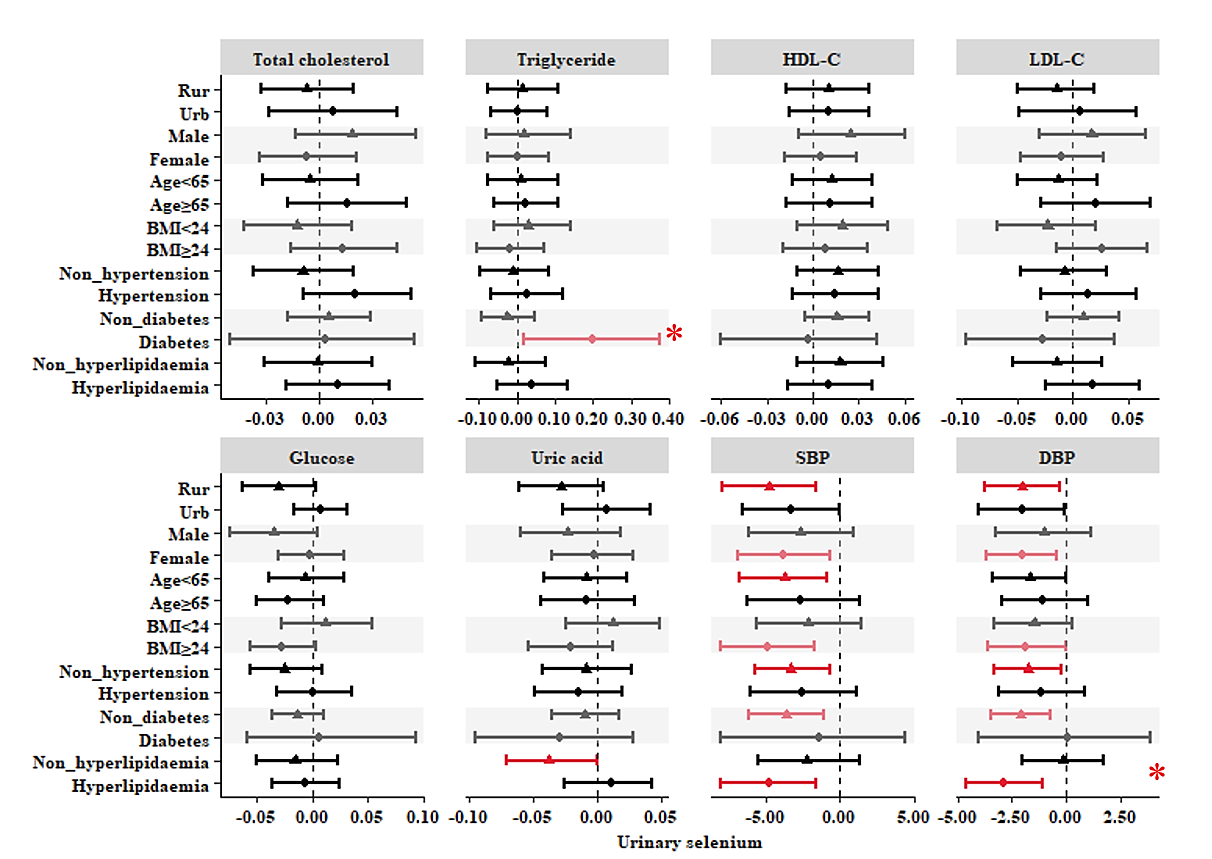
**

**Figure S7. Estimates and 95% confidence intervals of cardiometabolic risk factors associated with one-unit increase of natural log transformed urinary selenium, stratified by gender, age, BMI, hypertension status, diabetes status, dyslipidaemia status.**

Red indicates statistical significance (*p* < 0.05). Asterisk (*) indicates significant difference in subgroups.

Abbreviation: Rur: rural area; Urb: urban area; BMI: Body Mass Index; HDL-C: high-density lipoprotein cholesterol; LDL-C: low-density lipoprotein cholesterol; SBP: systolic blood pressure; DBP: diastolic blood pressure.


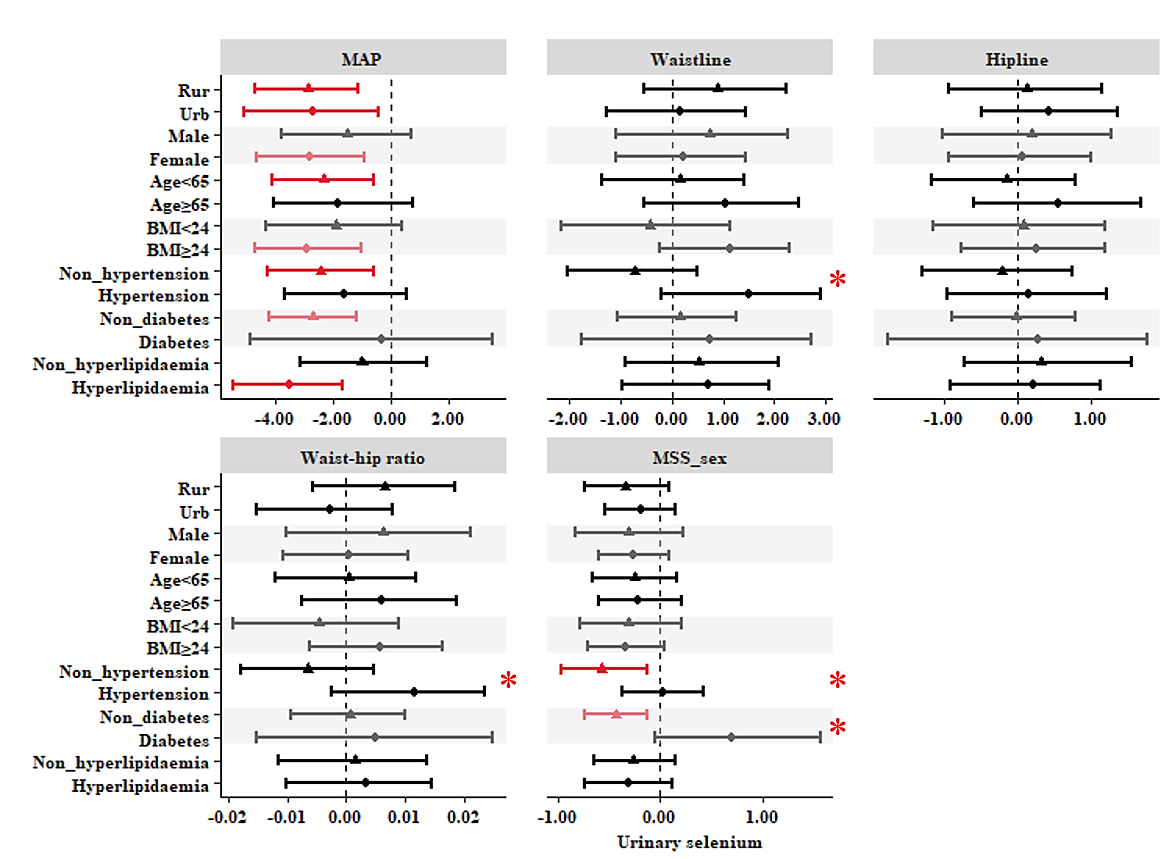


**Figure S7-continued. Estimates and 95% confidence intervals of cardiometabolic risk factors associated with one-unit increase of natural log transformed urinary selenium, stratified by gender, age, BMI, hypertension status, diabetes status, dyslipidaemia status.**

Red indicates statistical significance (*p* < 0.05). Asterisk (*) indicates significant difference in subgroups.

Abbreviation: Rur: rural area; Urb: urban area; MAP: mean arterial pressure; MSS-sex: metabolic syndrome score (sex specific).

**
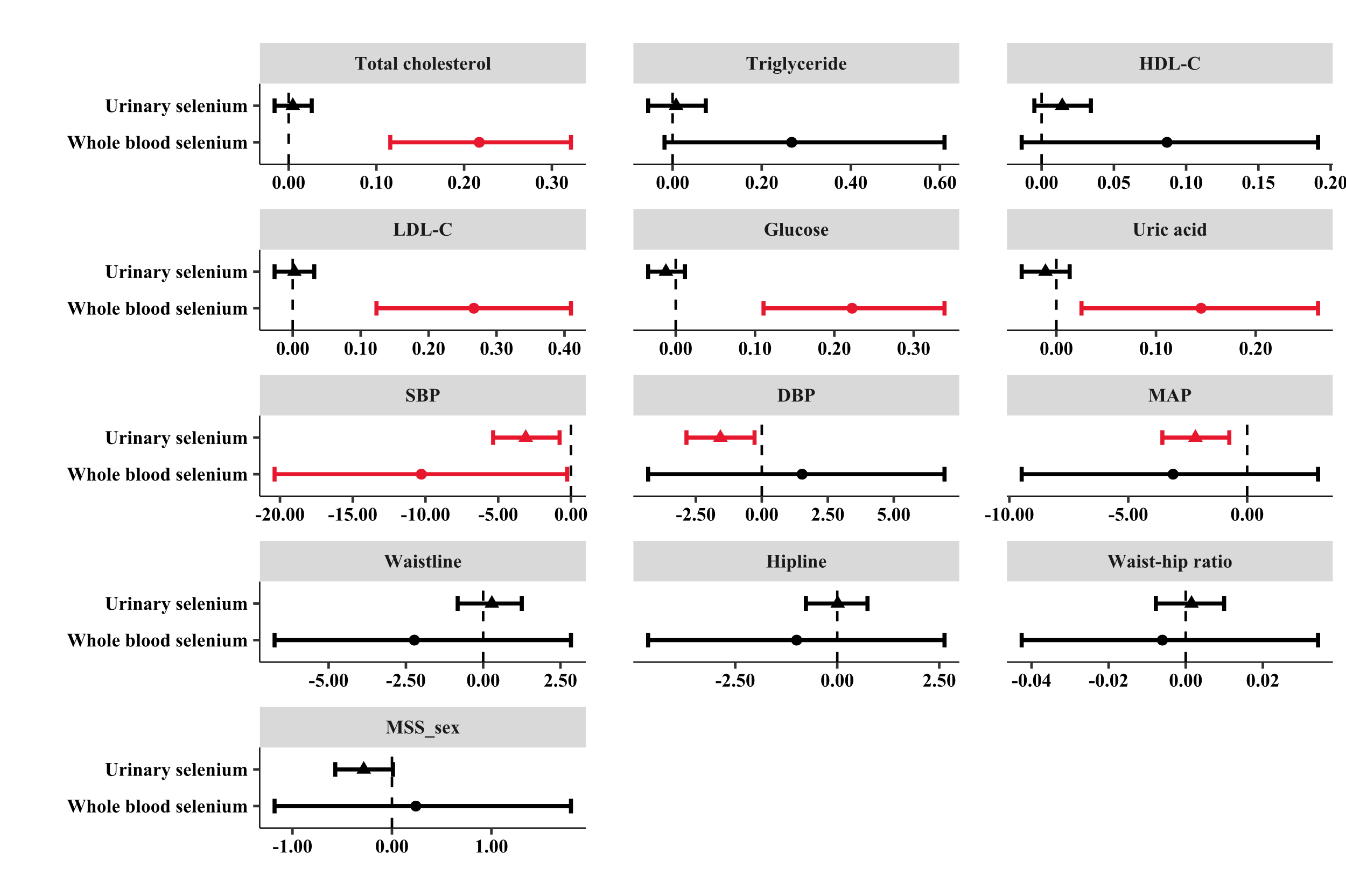
**

**Figure S8. Estimates and 95% confidence intervals of cardiometabolic risk factors associated with one-unit increase of natural log transformed selenium biomarkers, additionally adjusting for history of hypertension, diabetes, or dyslipidaemia on the basis of main model.**

Red indicates statistical significance (*p* < 0.05).

Abbreviation: BMI: Body Mass Index; HDL-C: high-density lipoprotein cholesterol; LDL-C: low-density lipoprotein cholesterol; SBP: systolic blood pressure; DBP: diastolic blood pressure; MAP: mean arterial pressure; MSS-sex: metabolic syndrome score (sex specific).


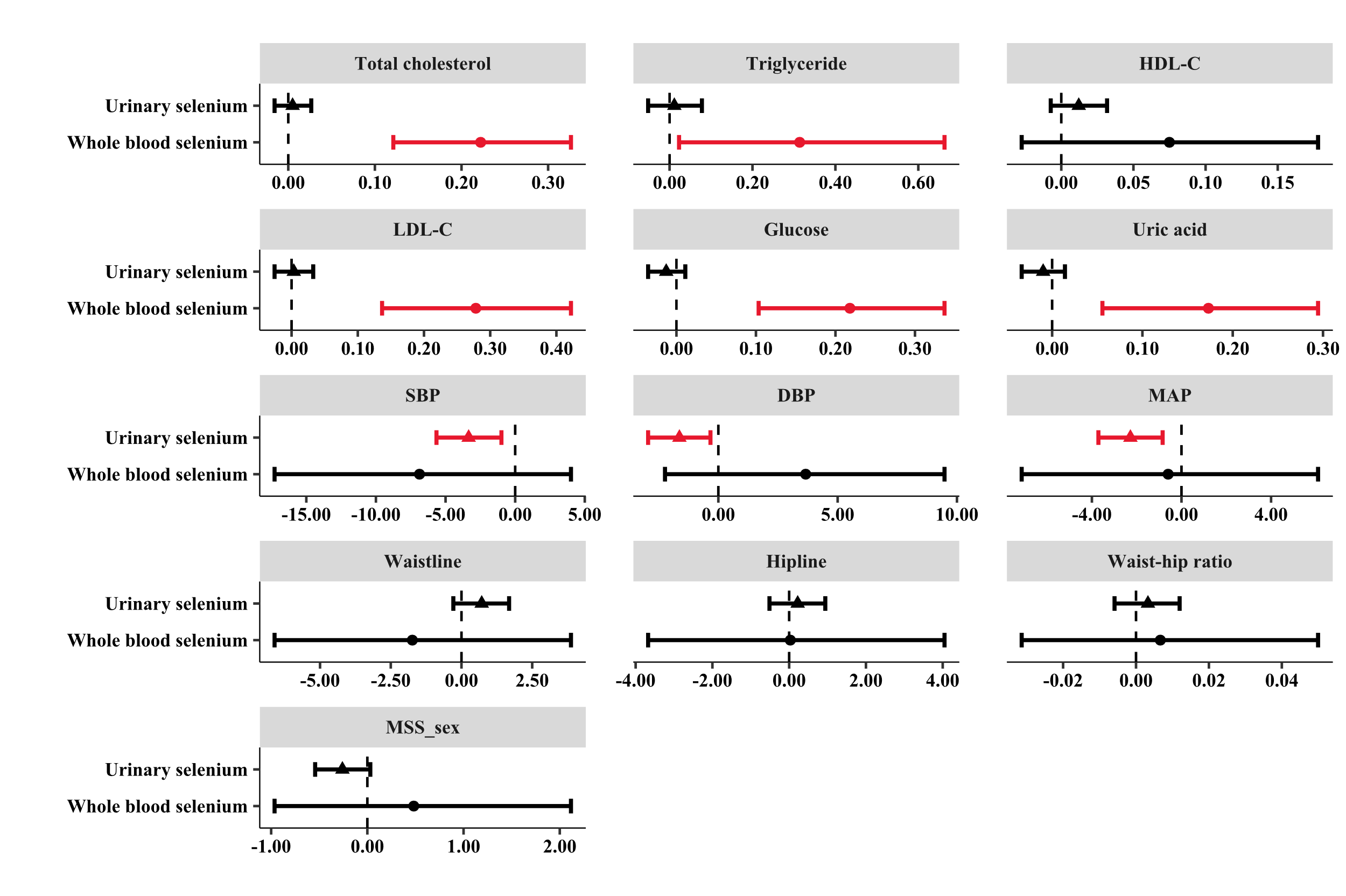


**Figure S9. Estimates and 95% confidence intervals of cardiometabolic risk factors associated with one-unit increase of natural log transformed selenium biomarkers, excluding BMI from covariates of main model.**

Red indicates statistical significance (*p* < 0.05).

Abbreviation: BMI: Body Mass Index; HDL-C: high-density lipoprotein cholesterol; LDL-C: low-density lipoprotein cholesterol; SBP: systolic blood pressure; DBP: diastolic blood pressure; MAP: mean arterial pressure; MSS-sex: metabolic syndrome score (sex specific).

**
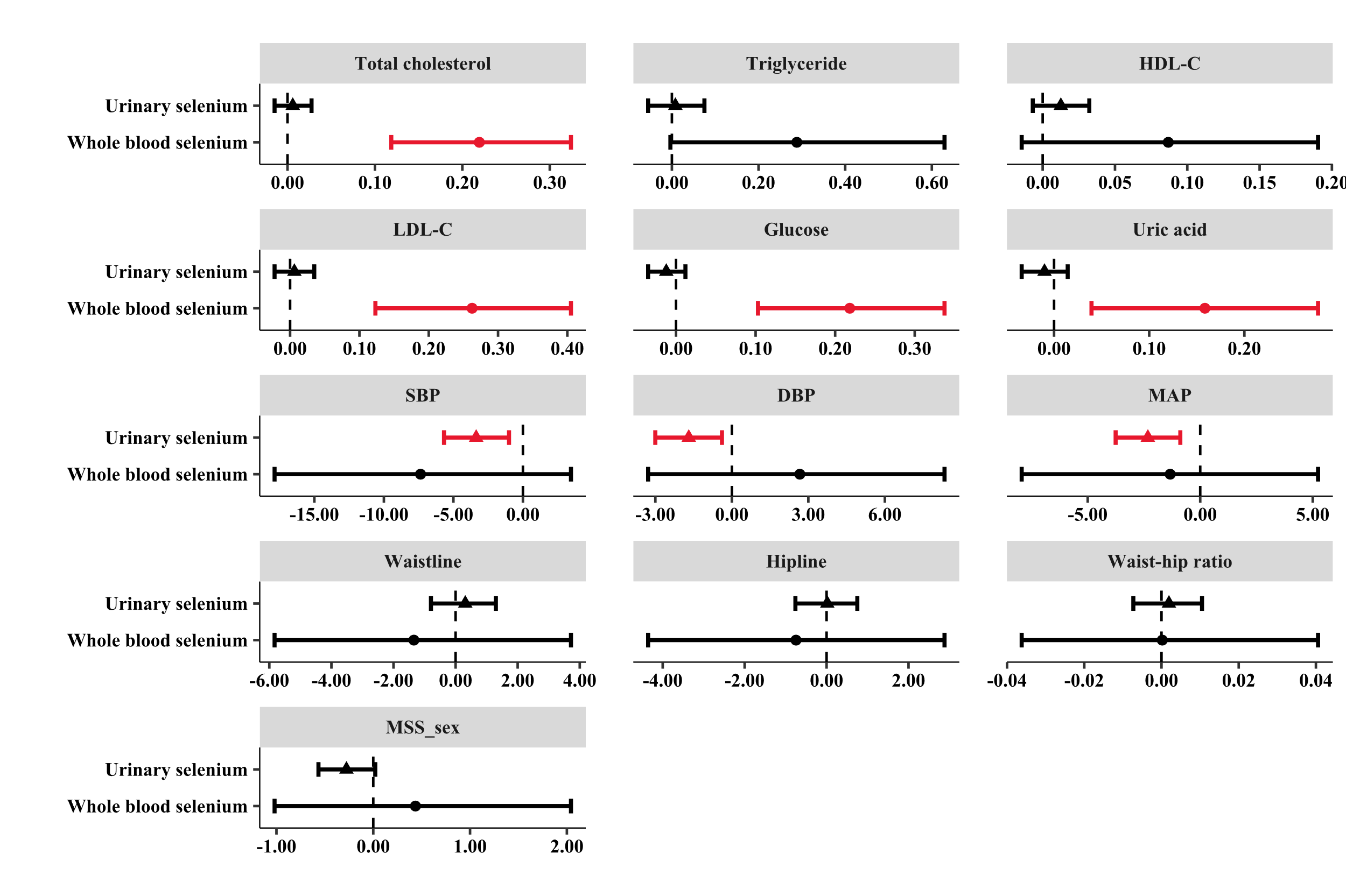
**

**Figure S10. Estimates and 95% confidence intervals of cardiometabolic risk factors associated with one-unit increase of natural log transformed selenium biomarkers, additionally corrected for high‐sensitivity C‐reactive protein on the basis of main model.**

Red indicates statistical significance (*p* < 0.05).

Abbreviation: BMI: Body Mass Index; HDL-C: high-density lipoprotein cholesterol; LDL-C: low-density lipoprotein cholesterol; SBP: systolic blood pressure; DBP: diastolic blood pressure; MAP: mean arterial pressure; MSS-sex: metabolic syndrome score (sex specific).
